# A conserved Arf-GEF modulates axonal integrity through RAB-35 by altering neuron–epidermal attachment

**DOI:** 10.1242/jcs.264565

**Published:** 2026-07-13

**Authors:** Igor Bonacossa-Pereira, Dat Le, Sean Coakley, Massimo A. Hilliard

**Affiliations:** ^1^ Clem Jones Centre for Ageing Dementia Research, Queensland Brain Institute, Faculty of Health, Medicine and Behavioural Sciences, The University of Queensland, Brisbane, Queensland 4072, Australia; ^2^ School of Biomedical Sciences, Faculty of Health, Medicine and Behavioural Sciences, The University of Queensland, Brisbane, Queensland, 4072, Australia

**Keywords:** AGEF-1, ARFGEF2, RAB-35, Axonal integrity, Spectrin, Attachment

## Abstract

Neurites of sensory neurons innervate the skin and are embedded within it. These delicate structures are chronically exposed to mechanical strain and yet their integrity is maintained throughout life. In *C. elegans,* UNC-70 (a β-spectrin protein) functions in synergy with the small GTPase RAB-35 within the skin to stabilize neuron–epidermal attachment against mechanical strain and prevent movement-induced damage to mechanosensitive axons. However, the molecular pathway regulating these specialized attachments remains elusive. Here, through an unbiased genetic screen, we have identified a guanine nucleotide exchange factor (GEF), AGEF-1, that impacts axonal maintenance. This molecule is known to function in endocytic recycling and its human ortholog, BIG2 (also known as ARFGEF2), is associated with the development of the periventricular nodular heterotopia. We show that AGEF-1 functions selectively within the skin to regulate axonal integrity of mechanosensitive neurons. Mechanistically, we reveal that AGEF-1 binds to epidermal RAB-35 and regulates its activity, modulating neuron–epidermal attachment stability. Finally, we demonstrate that this GEF is highly conserved, with its human ortholog BIG2 being capable of replacing AGEF-1. Together, we reveal the molecular machinery responsible for fine-tuning neuron–epidermal attachments and maintaining axonal integrity during life.

## Teaser

**Highlighted Article:** AGEF-1, as well as its human ortholog BIG2, is associated with recessive periventricular nodular heterotopia, as a modulator of axonal integrity in *C. elegans*.

## ADDITIONAL LANGUAGE ABSTRACT (BRAZILIAN PORTUGUESE)

Neuritos de neurônios sensoriais inervam densamente a pele e situam-se inseridos nela. Essas estruturas delicadas são cronicamente expostas a tensão mecânica, contudo sua integridade é mantida ao longo da vida. Em *C. elegans*, a β-espectrina UNC-70 atua em sinergia com a GTPase RAB-35 dentro da pele para estabilizar adesões neuro-epidermais contra tensão mecânica e protege axônios mecanosensitivos contra dano mediado pelo movimento. No entanto, não são conhecidos todos os componentes da maquinaria molecular que regula essas adesões. Através de uma mutagênese química, identificamos que AGEF-1, um fator de troca de nucleotídeos de guanina (GEF), impacta a manutenção axonal de neuronios mecanosensitivos. Essa molecula funciona no sistema de reciclagem endocítica e seu ortologo em humanos, BIG2, é associado ao desenvolvimento de heterotropia nodular periventricular. Aqui demostramos que essa proteína atua seletivamente na pele para essa função. Mecanisticamente, revelamos que AGEF-1 liga-se a RAB-35 epidermal regulando sua ativação e modulando a estabilidade de adesões neuro-epidermais. Por fim, demonstramos que AGEF-1 é altamente conservado, com o seu ortólogo humano BIG2 sendo capaz de substituir a molécula nematoide. Ao todo, revelamos a maquinária molecular responsável pelo ajuste delicado das adesões neuro-epidermais e da integridade axonal ao longo da vida.

## INTRODUCTION

Neurons extend disproportionately long axons that develop specialized attachments with surrounding cells. This is especially evident in sensory axons innervating the epidermis. Neuron–epidermal attachments induce the embedment of sensory axons within the skin, tightly adhering and mechanically coupling these tissues (Bonacossa-Pereira et al., 2022; Coakley et al., 2020; Jiang et al., 2019; Rosa et al., 2023; Yang et al., 2019). Owing to their slender architecture and anatomical localization, these axons are vulnerable to movement-induced mechanical strain (Bonacossa-Pereira et al., 2022; Coakley et al., 2020, 2026; Coppini et al., 2024; Krieg et al., 2017; Nekimken et al., 2020; Phillips et al., 2004).

Spectrins provide mechanical resistance and maintain membrane shape against strain in all cells (Bennett and Lorenzo, 2013). Axons are enriched in β-spectrin (D'Este et al., 2015; He et al., 2016; Xu et al., 2013), which contributes to axonal elasticity and force resistance (Bonacossa-Pereira et al., 2022; Coakley et al., 2026; Krieg et al., 2017). We have recently demonstrated, using the nematode *C. elegans* as a model system, that axonal β-spectrin is not sufficient to protect sensory neurons from damage, and instead functions non-cell-autonomously within the epidermis to maintain the integrity of the posterior lateral mechanosensory neuron (PLM) (Coakley et al., 2020, 2026). During development, these neurons attach to and embed within the epidermis via specialized neuron–epidermal attachments (Bonacossa-Pereira et al., 2022; Chalfie and Sulston, 1981). These attachments must be mechanically resilient to prevent damage to the PLM axon upon body movement. We have shown that the mechanical resilience of the neuron–epidermal attachments is mediated by β-spectrin and a small GTPase RAB-35, which function together in the epidermis to stabilize these attachments against movement and maintain axonal integrity (Coakley et al., 2020).

Small GTPases are molecular switches that cycle between active and inactive states to regulate various cellular pathways, including cytoskeleton remodeling and membrane recycling (Cherfils and Zeghouf, 2013). They function as molecular switches, cycling between an active GTP-bound state and an inactive GDP-bound state. GTPase-activating proteins (GAPs) stimulate the intrinsic GTPase activity and terminate effector binding, whereas guanine nucleotide exchange factors (GEFs) promote the active state by stimulating the release of GDP to allow GTP binding. Chronic activation of the epidermal small GTPase RAB-35, induced either by the loss of the GAP TBC-10 or by a mutation that renders RAB-35 constitutively active, causes catastrophic PLM axon breaks when the epidermal spectrin network is disrupted (Coakley et al., 2020). This supports a model where the regulation of RAB-35 activity is required, redundantly with epidermal β-spectrin, to maintain axonal integrity. Although two RAB-35-specific GEFs, RME-4 and FLCN-1, have been identified to activate RAB-35 in the context of endocytosis and cell corpse clearance (Kutscher et al., 2018; Sato et al., 2008), in the context of axonal maintenance RME-4 is only partially involved and FLCN-1 has no role (Coakley et al., 2020). This suggests that there are as-yet-unknown RAB-35 activators that function to regulate axonal maintenance.

To identify novel RAB-35 activators modulating axonal integrity, we performed an unbiased genetic screen for suppressors of axonal damage in sensitized animals where the PLM axon spontaneously breaks. From this screen, we identified AGEF-1, an Arf GEF previously associated with endocytic recycling and cell-attachment polarity pathways (Sheen, 2014; Shin et al., 2004; Skorobogata et al., 2014; Tang et al., 2012). We demonstrate that this molecular function is evolutionarily conserved with its human ortholog BIG2 (also known as ARFGEF2), which is capable of replacing the nematode gene function. Next, we reveal that AGEF-1 functions within the skin, in a non-cell-autonomous fashion, to modulate the integrity of the embedded axon. Finally, we show that AGEF-1 interacts with RAB-35 and regulates its activity *in vivo* to impact axonal integrity by altering the neuron-epidermal attachments.

## RESULTS

### AGEF-1/BIG2 regulate axonal integrity non-cell-autonomously in the epidermis

**Fig. 1.**
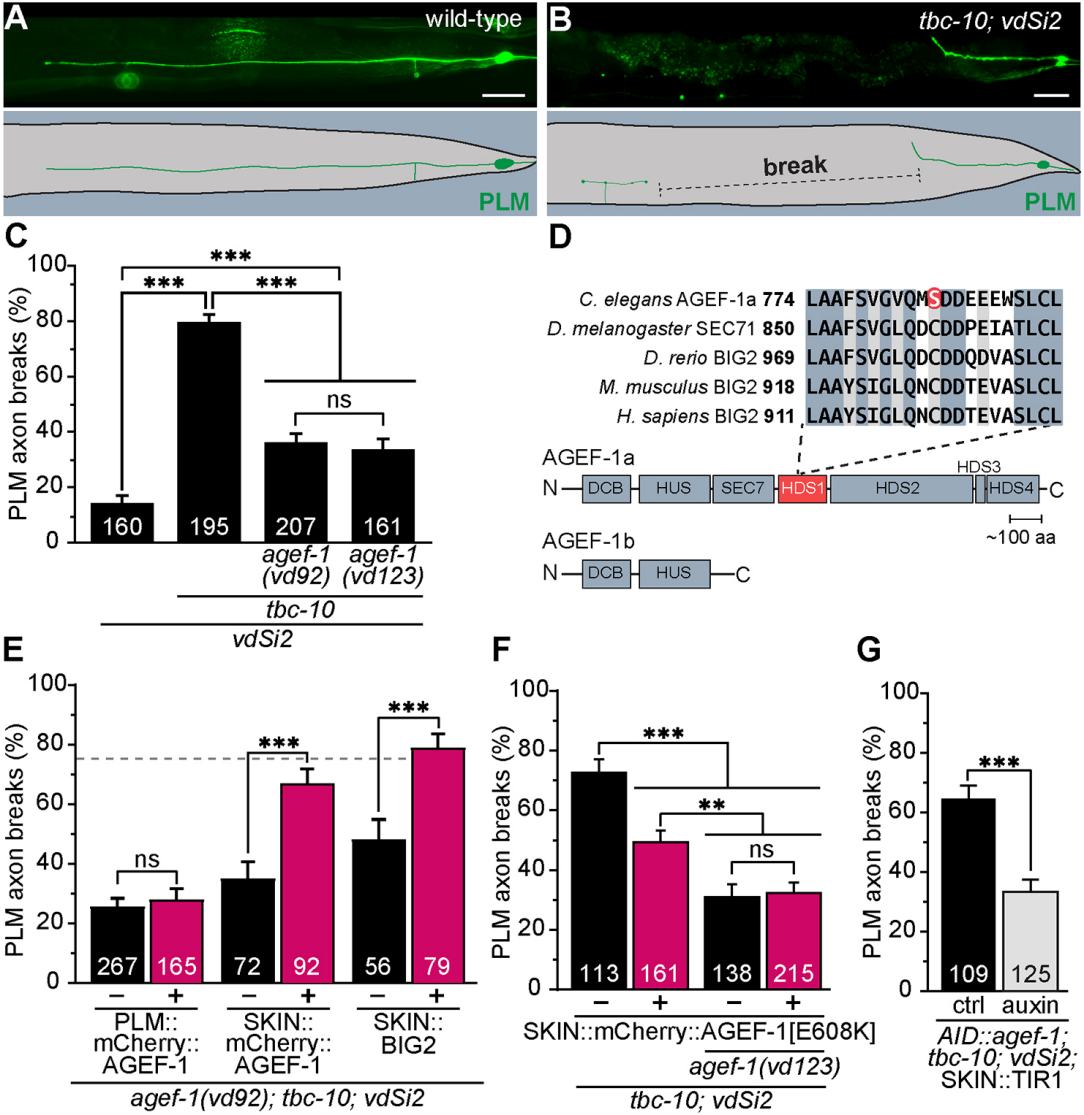
**AGEF-1 and BIG2 are conserved and function non-cell autonomously in the epidermis to modulate axonal integrity.** (A,B) Lateral view of the tail of adult animals with schematics of the observed axonal morphology. (A) Wild-type, intact PLM axon (Pmec-4::GFP). (B) *vdSi2; tbc-10* sensitized animal, showing a broken PLM axon. Scale bars: 20 μm. (C) Mean penetrance of PLM axon breaks in *vdSi2* single transgenics, *vdSi2; tbc-10* double mutants, *agef-1(vd92); tbc-10; vdSi2* triple mutants and CRIPSR-engineered *agef-1(vd123); tbc-10; vdSi2* triple mutants. (D) Predicted sequences encoded by *agef-1* highlighting the residue mutated in *agef-1(vd92)* and its corresponding orthologs. Numbering indicates residue position. Light blue, conserved residues; gray, equivalent residues; red, *vd92* mutation site. (E) Mean penetrance of PLM axon breaks in *agef-1(vd92); tbc-10; vdSi2* triple mutants expressing the *C. elegans* AGEF-1 cDNA selectively within the PLM neurons (PLM, *Pmec-4*) or skin (SKIN, *Pdpy-7*), and human BIG2 cDNA selectively in the skin. Dashed line indicates the phenotype of *vdSi2; tbc-10* sensitized animals. (F) Mean penetrance of PLM axon breaks in *vdSi2; tbc-10* and *agef-1(vd123); tbc-10; vdSi2* animals expressing a dominant-negative *C. elegans* AGEF-1[E698K] cDNA tissue-specifically in the skin. Magenta bars represent transgenic animals and black bars their non-transgenic siblings. (G) Mean penetrance of PLM axon breaks in *vdSi2; tbc-10* expressing TIR1 selectively in the skin (SKIN::TIR1) when raised in the presence of auxin or on control plates (ctrl) with regular NGM agar. Black bar highlights controls. Gray bar highlights auxin group. A represents the mean penetrance based on a uniform-prior distribution. All error bars show mean±s.e.m. Sample size represented within the bars. ***P<0.001; **P<0.01; ns, not significant (one-way ANOVA followed by Tukey's multiple comparisons was used to compare multiple groups; unpaired, two-tailed *t*-test used to compare transgenics versus non-transgenics).

Small GTPase activation by GEFs is a highly contextual and redundant mechanism, thus predicting the GEFs relevant for a given functional outcome is challenging (Cherfils and Zeghouf, 2013). To identify novel GEFs relevant to axonal integrity, we conducted an unbiased forward genetic screen in a sensitized genetic background in which sensory axons spontaneously break due to mechanical strain. Specifically, we used animals expressing a dominant negative *unc-70* cDNA (*unc-70* encodes a *C. elegans* β-spectrin) selectively within the epidermis [denoted as the *vdSi2* genotype, the full genotype of which is *SKIN::UNC-70(n493)::mKate*] combined with a loss-of-function allele of *tbc-10,* a GAP that inactivates RAB-35 (genotype, *tbc-10; vdSi2*) (Coakley et al., 2020; Kutscher et al., 2018; Sato et al., 2008). In these animals the PLM neuron and axon develop normally, but the axon spontaneously breaks and degenerates in adults (Coakley et al., 2020) (Fig. 1A–C). From this screen, we identified the recessive mutation *vd92*, which caused a strong suppression of PLM axon breaks (Fig. 1C). Whole-genome sequencing and direct mapping (Zuryn et al., 2010) of *vd92* revealed a missense mutation in the *agef-1* locus, which encodes the long AGEF-1a isoform (hereafter referred to as AGEF-1) that contains a catalytic GTP-exchange domain (SEC7), and is predicted to encode a truncated version, AGEF-1b, which lacks the SEC7 domain and downstream regulatory domains (Fig. 1D) (Sternberg et al., 2024). AGEF-1 is an evolutionarily conserved GEF proposed to activate adenosine diphosphate-ribosylation factor (Arf) GTPases implicated in the endocytic-recycling pathway (McDonold and Fromme, 2014; Sato et al., 2006; Skorobogata et al., 2014; Tang et al., 2012). The *vd92* mutant allele carried a serine to leucine substitution in amino acid 784 (S784L), which is located within the ‘homology downstream of SEC7’ domain (HDS1) of AGEF-1, downstream of the SEC7 domain (Fig. 1D; Fig. S1A). To confirm that *vd92* is an allele of *agef-1*, we replicated this mutation using CRISPR-Cas9 to engineer the mutant allele *agef-1(vd123).* Indeed, we observed that *agef-1(vd123)* phenocopied *agef-1(vd92)* in suppressing PLM axon breaks in *tbc-10; vdSi2* animals; as predicted, this mutation did not induce PLM axon breaks nor any other detectable phenotype when in a wild-type background (Fig. S1B). Taken together, these results conclusively reveal that *vd92* is an allele of *agef-1*.

Next, to determine the tissue in which *agef-1* functions to regulate axonal integrity we selectively expressed a wild-type copy of the AGEF-1 cDNA with an N-terminal mCherry tag in either the epidermis (*SKIN::mCherry::AGEF-1*) or the mechanosensory neurons (*PLM::mCherry::AGEF-1*), in *agef-1(vd92); tbc-10; vdSi2* mutant animals. We found that only *SKIN::mCherry::AGEF-1* was able to rescue the mutant phenotype and restore the rate of PLM axon breaks to background levels (Fig. 1E). Thus, AGEF-1 functions selectively within the epidermis to regulate PLM axon integrity, in a similar fashion to RAB-35, TBC-10 and UNC-70 (Coakley et al., 2020).

*ARFGEF2* is the human ortholog of *agef-1* and encodes the brefeldin A-inhibited guanine nucleotide-exchange protein 2 (BIG2); although the human and nematode proteins share a high degree of amino acid sequence conservation, the serine residue mutated in *agef-1(vd92)* is not conserved (Fig. 1D). To determine whether AGEF-1 is functionally conserved, we expressed wild-type human BIG2 cDNA selectively within the epidermis (*SKIN::BIG2*) of *agef-1(vd92); tbc-10; vdSi2* animals to determine its capacity to compensate for the loss of AGEF-1 function. Importantly, we found that *SKIN::BIG2* fully restored the penetrance of PLM axon breaks (Fig. 1E), supporting the notion that the molecular function of AGEF-1 in mediating axonal integrity is conserved. We also attempted to generate a full deletion of the *agef-1* locus using CRISPR-Cas9, but the resulting deletion was lethal, consistent with previous reports that *agef-1* is required for viability (Skorobogata et al., 2014). This indicated that the *agef-1(vd92)* is not a null, but likely a loss-of-function allele. Thus, we next decided to investigate the functional consequence of the S784L mutation. Given that this mutation falls within a regulatory domain (McDonold and Fromme, 2014; Richardson et al., 2012) (Fig. 1D), we hypothesized that it might affect the GEF function. In this model, if this function is required for axonal damage then overexpression of a dominant negative, catalytically inactive AGEF-1 should phenocopy the effect of *agef-1(vd92)*. To test this notion, we engineered a dominant negative *agef-1* cDNA (AGEF-1[E608K]), which mimics a previously validated mutation in BIG2 that renders the SEC7 domain catalytically inactive (Shinotsuka et al., 2002b). We then overexpressed this mutated gene within the epidermis (*SKIN::mCherry::AGEF-1[E608K]*) of *tbc-10; vdSi2* animals, and found that it suppressed the PLM axon breaks, phenocopying *agef-1(vd123); tbc-10; vdSi2* animals (Fig. 1F). Moreover, we observed that overexpression of *SKIN::mCherry::AGEF-1[E608K]* had no effect on *agef-1(vd123); tbc-10; vdSi2* mutants (Fig. 1F). Finally, to conclusively test whether *agef-1(vd92)* is a loss-of-function allele, we used an auxin-inducible degradation (AID) system (Ashley et al., 2021) to tissue-specifically degrade endogenous AGEF-1 in the skin of *vdSi2; tbc-10* animals. To do this, we N-terminally tagged endogenous *agef-1* with an AID degron sequence (*AID::agef-1*) using CRISPR-Cas9 and expressed the *Arabidopsis thaliana* F-box transport inhibitor response 1 protein (TIR1) selectively in the skin (SKIN::TIR1). Skin-specific degradation of AGEF-1 in *AID::agef-1; tbc-10; vdSi2* animals phenocopied *agef-1(vd92); tbc-10; vdSi2*, suppressing PLM axon breaks (Fig. 1G). Thus, we conclude that *agef-1(vd92)* is a partial loss-of-function allele and, although the S784L mutation falls in a regulatory domain, ultimately it impairs GEF function.

In summary, these results indicate that AGEF-1 functions within the epidermis to regulate the integrity of the PLM axon through the activation of GTPases, and that this molecular function is conserved in the human ortholog BIG2.

### AGEF-1 functions upstream of RAB-35

AGEF-1/BIG2 is known to participate in the endocytic-recycling pathway in multiple cellular contexts by physically binding and activating Arf GTPases, particularly ARF-1 and ARF-5 (Lovrecic et al., 2010; Shinotsuka et al., 2002a; Skorobogata et al., 2014; Tang et al., 2012); however, it is not clear whether Arf GTPases are its exclusive targets (Sato et al., 2006; Shinotsuka et al., 2002b). Given the genetic context in which we identified *agef-1,* we hypothesized that AGEF-1 could facilitate the activation of the GTPase RAB-35 to regulate axonal integrity. This could be a direct mechanism that requires AGEF-1 to bind RAB-35, or an indirect mechanism that requires activation of another GTPase, or a suite of GTPases, that in turn potentiate the activation of RAB-35, as observed in the context of tumor migration (Kulasekaran et al., 2021). To address this notion, we took a multifaceted approach.

**Fig. 2.**
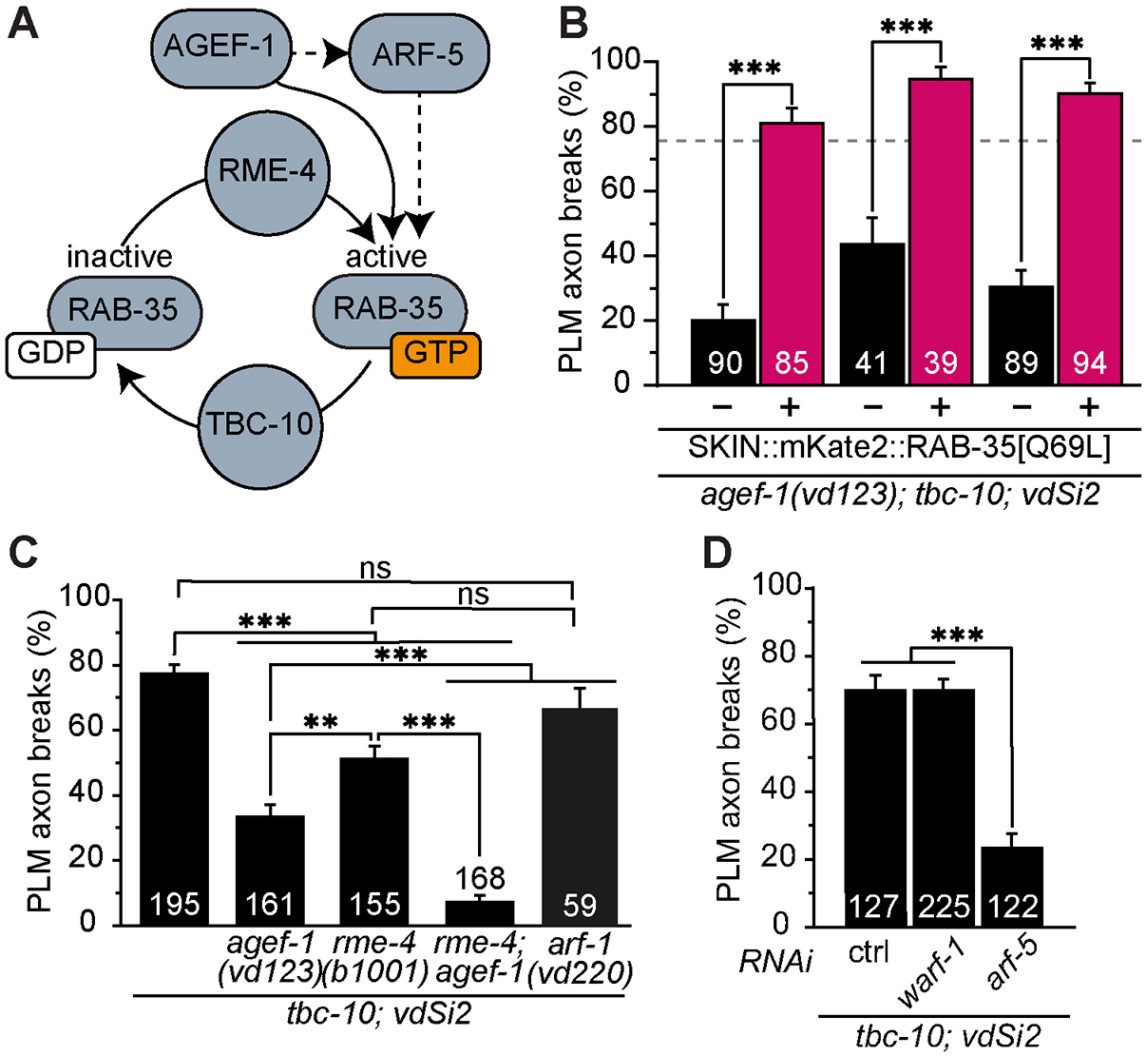
**AGEF-1 functions upstream of RAB-35 activity.** (A) Schematic of the RAB-35 activation cycle. Solid lines indicate directly validated gene interactions; dashed lines indicate predicted genetic interactions. (B) Mean penetrance of PLM axon breaks in three independent transgenic lines of *agef-1(vd123); tbc-10; vdSi2* triple mutants expressing a constitutively active mKate2::RAB-35[Q69L] tissue-specifically within the skin (SKIN, *Pdpy-7*). Dashed line indicates the phenotype of *vdSi2; tbc-10* sensitized animals. Magenta bars represent transgenic animals and black bars their non-transgenic siblings. (C) Mean penetrance of PLM axon breaks in *tbc-10*; *vdSi2* sensitized animals, *agef-1(vd123); tbc-10; vdSi2* triple mutants, *rme-4(b1001); tbc-10; vdSi2* triple mutants, *rme-4(b1001); agef-1(vd123); tbc-10; vdSi2* quadruple mutants and *arf-1(vd220); tbc-10; vdsi2* triple mutants. (D) Mean penetrance of PLM axon breaks in *vdSi2; tbc-10* sensitized animals grown in HT115 *E. coli* containing an empty vector, a *warf-1* RNAi encoding vector or an *arf-5* RNAi encoding vector. Bars represent the mean penetrance based on a uniform-prior distribution. All error bars show mean±s.e.m. Sample size represented within the bars. ***P<0.001; **P<0.01; ns, not significant (one-way ANOVA followed by Tukey's multiple comparisons was used to compare multiple groups; unpaired, two-tailed *t*-test used to compare transgenics versus non-transgenics).

First, we reasoned that if AGEF-1 functions in RAB-35 activation, it must act upstream of active, GTP-bound RAB-35 (Fig. 2A). To test this possibility, we overexpressed a dominant, constitutively active RAB-35 in the epidermis of *agef-1(vd123); tbc-10; vdSi2* animals (*SKIN:: RAB-35[Q69L]*). We found that constitutively active RAB-35 fully restored the penetrance of PLM axon breaks in *agef-1(vd123); tbc-10; vdSi2* compared to their non-transgenic siblings (three independent lines, Fig. 2B), consistent with *agef-1* functioning upstream of active GTP-bound RAB-35*.*

Next, we tested whether AGEF-1 functions in synergy with another known activator of RAB-35 (Fig. 2A). To do this, we determined the genetic interaction between *agef-1* and *rme-4,* a known RAB-35 GEF that partially modulates axonal integrity (Coakley et al., 2020; Sato et al., 2008). In *tbc-10; vdSi2* animals, the strong loss-of-function allele *rme-4(b1001)* (Sato et al., 2008) partially suppresses PLM axon breaks, although less effectively than *agef-1(vd123)* (Fig. 2C and Coakley et al., 2020). In contrast, *rme-4; agef-1; tbc-10; vdSi2* animals displayed a full suppression of PLM axon breaks (Fig. 2C), supporting the notion that these molecules function redundantly.

Given that BIG2 is known to activate Arf1, Arf3 and Arf5 (Shin et al., 2004), we next tested whether the nematode orthologs of these GTPases would affect PLM axon breaks. We reasoned that if this was the case, the loss of these molecules would suppress PLM breaks in *tbc-10; vdSi2* animals. We used a combination of CRISPR-Cas9-induced knockout and RNA-interference (RNAi) knockdown (Timmons et al., 2001) of target genes to test this notion. Disruption of the Arf1 orthologs, via knockout of *arf-1* and knockdown of *warf-1*, had no effect on PLM breaks (Fig. 2C,D). In contrast, despite the highly penetrant larval lethality and vulval development defects, *arf-5* knockdown suppressed PLM breakage in *tbc-10; vdSi2* survivors, mimicking the effect of *agef-1(vd92)* (Fig. 2D). These results indicate that ARF-5 (the nematode ortholog of Arf3 and Arf5) also regulate axonal integrity.

Taken together, our data supports a model whereby AGEF-1 functions upstream of active RAB-35 in redundance with RME-4, and that ARF-5 possibly functions upstream of active RAB-35.

### AGEF-1 interacts with RAB-35 in the epidermis

**Fig. 3.**
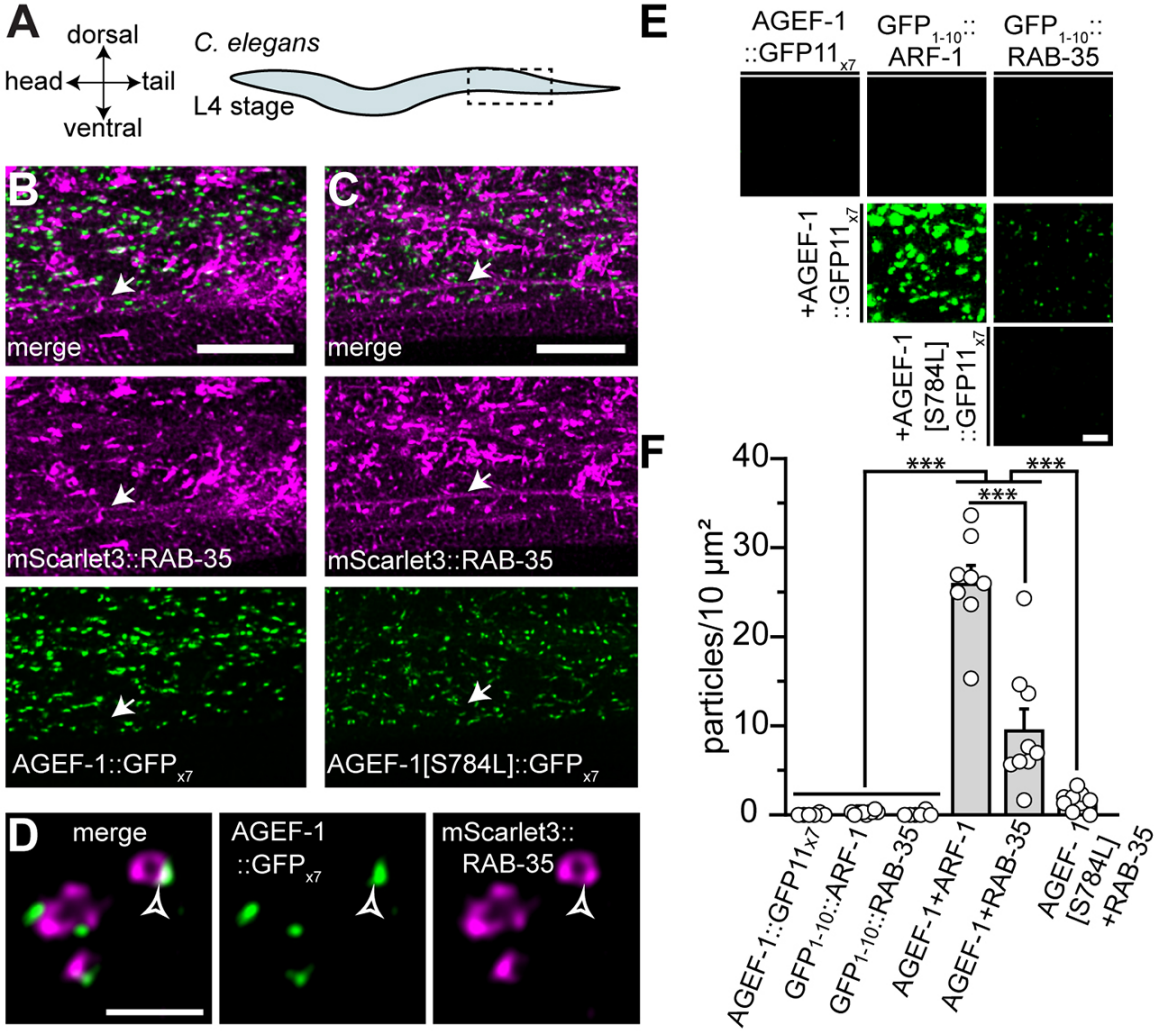
**AGEF-1 interacts with RAB-35 *in vivo* within the epidermis.** (A) Schematic of a lateral view of the animal highlighting the anatomical region shown in the B and C. (B) Representative maximum intensity projection of a deconvolved confocal microscopy of the epidermis of an engineered animal expressing endogenous mScarlet3::RAB-35 and endogenous epidermal AGEF-1::GFP_x7_ (AGEF-1::GFP_11×7_+SKIN::GFP_1-10_). (C) Representative maximum intensity projection of a deconvolved confocal microscopy of the epidermis of an engineered animal expressing endogenous mScarlet3::RAB-35 and endogenous epidermal AGEF-1[S784L]::GFP_x7._ Scale bars: 10 μm. Arrows point to the epidermal membrane adjacent to the PLM neuron axon. (D) Representative maximum intensity projection of a deconvolved array-detector super-resolution microscopy of mScarlet3::RAB-35 and endogenous epidermal AGEF-1::GFP_x7_ structures. Arrowhead highlights region of colocalization between AGEF-1 and RAB-35. Scale bar: 2 μm. Images in B and C represent six experimental repeats for wild-type and seven for AGEF-1[S784L]; D is representative of seven experimental repeats. (E) Representative maximum intensity projection of a deconvolved confocal microscopy of GFP reconstitution in engineered animals expressing endogenous GFP_1-10_::ARF-1, alone or in combination with endogenous epidermal AGEF-1::GFP_11×7_, of animals expressing endogenous GFP_1-10_::RAB-35 alone or in combination with endogenous AGEF-1::GFP_11×7_ or AGEF-1[S784L]::GFP_11×7_ and of animals expressing AGEF-1::GFP11_×7_ alone. Scale bar: 1 μm. (F) Quantification of the average number of GFP-positive puncta in engineered animals expressing endogenous AGEF-1::GFP_11×7_, GFP_1-10_::ARF-1, GFP_1-10_::RAB-35 individually and combinations of AGEF-1::GFP_11×7_+GFP_1-10_::ARF-1, AGEF-1::GFP_11×7_+GFP_1-10_::RAB-35 and AGEF-1[S784L]::GFP_11×7_+GFP_1-10_::RAB-35. Error bars are mean±s.e.m.; white circles represent individual samples. ***P<0.001 (one-way ANOVA followed by Tukey's multiple comparisons was used to compare multiple groups).

Given our genetic data, we hypothesized that AGEF-1 could be interacting directly with RAB-35. Thus, we investigated the subcellular localization of AGEF-1 and RAB-35 within the epidermis. We leveraged a split-fluorophore strategy (Goudeau et al., 2021) to visualize endogenous AGEF-1 within the epidermis, using CRISPR-Cas9 to fuse seven tandem repeats of GFP_11_ with the C-terminus of endogenous AGEF-1 (*agef-1(vd124)*, which encodes AGEF-1::GFP_11×7_). We then expressed GFP_1-10_ under an epidermal-specific promoter (SKIN::GFP_1-10_) to reconstitute full-length GFP selectively within the epidermis (epidermal AGEF-1::GFP_x7_). To address the effect of the S784L mutation, we replicated this mutation in *agef-1(vd124)* animals to generate the mutant allele *agef-1(vd142[AGEF-1[S784L]::GFP_11×7_])*. Next, we visualized RAB-35 by using CRISPR-Cas9 to knockin mScarlet3 at the N-terminus of endogenous RAB-35. Using a combination of confocal and super-resolution microscopy, we observed the localization of these molecules *in vivo* in the last larval stage (L4; Fig. 3A). We found that epidermal AGEF-1::GFP_x7_ and AGEF-1[S784L]::GFP_x7_ localized to small puncta throughout the cytoplasm and membrane of the epidermis (Fig. 3B,C). We did not observe any enrichment of these molecules in the vicinity of the PLM axon, with both mutant and wild-type appearing morphologically identical (Fig. 3B,C). mScarlet3::RAB-35, however, localized to punctate, web-like and circular structures within the epidermis and was notably enriched at the epidermal furrow surrounding the PLM axon (Fig. 3B,C and Coakley et al., 2020). In both mutant and wild-type *agef-1* animals, mScarlet3::RAB-35 localization appeared morphologically identical with no change in expression (Fig. 3B,C; Fig. S2A). Although AGEF-1 and RAB-35 appeared to largely not colocalize, we noted that a subset of epidermal AGEF-1::GFP_x7_ puncta decorated mScarlet3::RAB-35 structures; super-resolution imaging of such regions revealed that AGEF-1::GFP_x7_ overlaps with the edge of these mScarlet3::RAB-35 circular structures (Fig. 3D).

Given these observations, we wondered whether a subset of these molecules physically interact. AlphaFold 3 modeling of the interaction between both molecules suggests that RAB-35 could bind to AGEF-1 directly through the HDS1 domain (Fig. S1A). To approach this question in a quantitative manner and in the relevant cellular context, we designed an *in vivo* GFP complementation assay (Bignon et al., 2022; Romei and Boxer, 2019). This consisted of using CRISPR-Cas9 to fuse split GFP_1-10_ to the N-terminus of endogenous RAB-35 in either the AGEF-1::GFP_11×7_ or AGEF-1[S784L]::GFP11_×7_ strains used in the subcellular localization analysis, and visualize full length GFP reconstitution to detect their proximity to one another. As a positive control, we also generated endogenous GFP_1-10_::ARF-1, a known AGEF-1 interactor, in the AGEF-1::GFP_11×7_ strain. We then quantified the number of GFP-positive particles near to the PLM axon using an automated counting method. In the absence of their binding partner, each molecule did not emit any detectable signal (Fig. 3F). Strikingly, we observed GFP reconstitution in AGEF-1::GFP_11×7_+GFP_1-10_::RAB-35 animals, although not to the same extent, or with the same localization pattern, as in the AGEF-1::GFP_11×7_+GFP_1-10_::ARF-1 control animals or AGEF-1::GFP_x7_ (Fig. 3E,F; Fig. S2B), corroborating the notion that only a subset of AGEF-1 interacts with RAB-35 in the epidermis. Importantly, AGEF-1[S784L]::GFP_11×7_+GFP_1-10_::RAB-35 animals could not reconstitute GFP (Fig. 3E,F). The magnitude of this effect (∼97% reduction) could not be explained by a reduction of RAB-35 expression levels (Fig. 3B,C; Fig. S2A; no reduction), or the mild reduction of AGEF-1[S784L] localized in puncta the skin compared to their wild-type counterparts (Fig. 3B,C; Fig. S2C; ∼23% reduction). Notably, we were unable to test AGEF-1[S784L]::GFP_11×7_+GFP_1-10_::ARF-1 animals given that the resulting strain was a synthetic lethal. These results demonstrate that a subset of AGEF-1 is in proximity to RAB-35 *in vivo* suggesting that these molecules interact, either directly or indirectly, and that the S784 residue in AGEF-1 is necessary for this interaction.

### AGEF-1 alters neuron–epidermal attachments

**Fig. 4.**
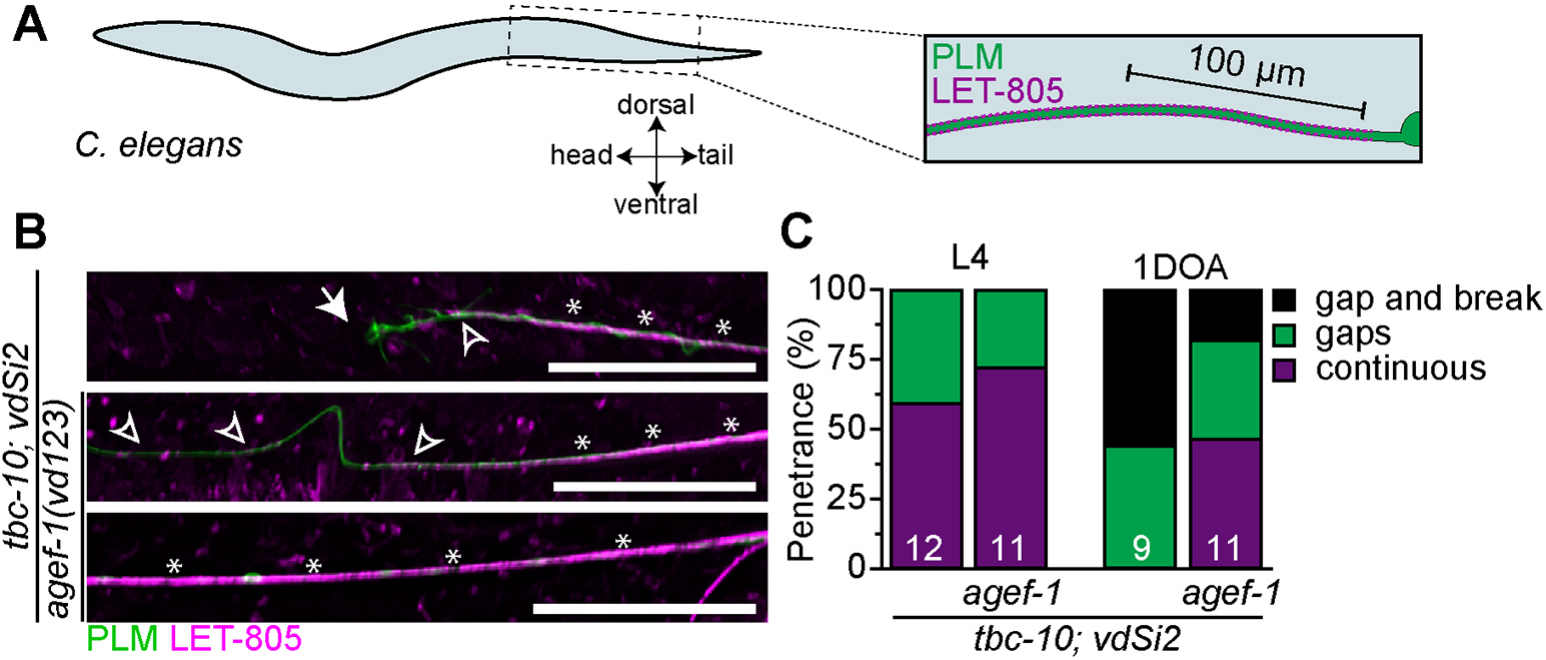
**AGEF-1 alters neuron–epidermal attachment.** (A) Schematic a lateral view of the animal highlighting the anatomical region analyzed. (B) Representative maximum intensity projection images of the quantified phenotypes, showing a lateral view of the PLM axon and endogenous LET-805::wrmScarlet. Top panel represents a *tbc-10; vdSi2* animal with the ‘gap and break’ phenotype. Middle panel represents an *agef-1(vd123); tbc-10; vdSi2* triple mutant animal with the ‘gaps’ phenotype. Bottom panel represents an *agef-1(vd123); tbc-10; vdSi2* triple mutant with the ‘continuous’ phenotype. Arrow highlights a PLM axon break. Arrowheads highlight naked PLM axons. Asterisks highlight regions of consistent neuron-epidermal attachment. Scale bars: 20 μm. (C) Quantification of the phenotypes observed in B showing the proportion of *tbc-10; vdSi2* sensitized animals and *agef-1(vd123); tbc-10; vdSi2* triple mutants at the last larval stage (L4) and 1-day-old adult (1DOA) stage. Sample size represented within the bars.

Correct development and maintenance of uniform neuron–epidermal attachments is essential to shield the PLM axon from breakage induced by the mechanical stress of body movement (Bonacossa-Pereira et al., 2022; Coakley et al., 2020). RAB-35 activity fine-tunes the resiliency of these specialized attachments in synergy with the epidermal β-spectrin protein UNC-70, with the appearance of discontinuous neuron–epidermal attachments after movement being predictive of axonal damage (Coakley et al., 2020). We next asked whether AGEF-1 also affects neuron–epidermal attachments via RAB-35. If AGEF-1 functions upstream of RAB-35 to regulate its activity, we predicted that AGEF-1 loss-of-function would protect neurons by suppressing the neuron–epidermal attachment defects present in *tbc-10; vdSi2* animals. To investigate this aspect, we observed the localization of a key epidermal attachment molecule, LET-805, which is part of the neuron–epidermal attachment complex (Bonacossa-Pereira et al., 2022; Chalfie and Sulston, 1981; Emtage et al., 2004). To visualize this molecule, we engineered transgenic animals in which the endogenous *let-805* locus is fused with the sequence encoding the fluorophore wrmScarlet (Bonacossa-Pereira et al., 2022; Coakley et al., 2020). We then analyzed the localization of LET-805::wrmScarlet in *agef-1(vd123); tbc-10; vdSi2* animals in comparison with *tbc-10; vdSi2* during the last larval stage (L4), and after the appearance of PLM axon breaks (1DOA) (Fig. 4A,B). We found that at the L4 stage, *agef-1(vd123); tbc-10; vdSi2* animals did not significantly differ from *tbc-10; vdSi2* animals (Fig. 4C). However, in adults all *tbc-10; vdSi2* animals presented a discontinuous LET-805::wrmScarlet localization along the PLM axon vicinity, with most of them accompanied by a PLM axon break (Fig. 4B,C). In stark contrast, approximately half of the *agef-1(vd123); tbc-10; vdSi2* animals retained a continuous LET-805::wrmScarlet localization (Fig. 4B,C). Of the adult *agef-1(vd123); tbc-10; vdSi2* animals that displayed gaps in LET-805::wrmScarlet localization near the PLM axon region, ∼67% displayed an intact axon (however, notably buckled), whereas the remaining ∼33% were broken (Fig. 4B,C).

Together, these results conclusively demonstrate that AGEF-1 loss-of-function suppresses the neuron–epidermal attachments defect present in *tbc-10; vdSi2* animals, protecting the associated sensory axons from movement-induced damage.

## DISCUSSION

By leveraging the power of unbiased forward genetics, we reveal that AGEF-1 regulates the axonal integrity of mechanosensory neurons in *C. elegans* by interacting with RAB-35 in the epidermis to modulate the stability of neuron–epidermal attachments in parallel to UNC-70 (Fig. S3). Furthermore, we demonstrate the evolutionary conservation of this molecular function by showing that the human ortholog BIG2 can replace AGEF-1 in the context of axonal maintenance. Importantly, our results highlight a trans-tissue maintenance mechanism whereby the integrity of mechanosensory neurons are regulated by components functioning non-cell autonomously within the epidermis. Finally, our results support a model where uniform neuron–epidermal attachments are tightly regulated to preserve the integrity of axons.

Variants in BIG2 are associated with the development of periventricular nodular heterotropia, and disruption of this molecule causes neuronal migration defects in culture models (Grzmil et al., 2010; Sheen et al., 2004; Tanyalçin et al., 2013)*.* The mechanism of action of BIG2 in neuronal health is unknown and, in general, research in this area focuses on neuron-intrinsic functions of this molecule. Here we show that AGEF-1/BIG2 can function non-cell-autonomously outside the nervous system to regulate axonal integrity, and that this molecular role is conserved. Although we have not tested the role of BIG1 (also known as ARFGEF1), we predict that our findings could also apply to this molecule, given the high structural and sequence conservation of BIG1 and BIG2. Future investigations of BIG1- and BIG2-associated neurological disorders should consider a non-neuronal function and hyperactivity of these proteins as a driver of neuropathology. Moreover, given that we observe a deleterious function of AGEF-1 and BIG2 that contributes to axonal damage in the context of a spectrin disruption and chronic RAB-35 over-activation, we propose that variants in BIG1 and BIG2 affecting its GEF activity could be disease-modifiers in individuals with spectrinopathies.

To our surprise, AGEF-1 functions independently of its classical target GTPase ARF-1 in the context of axonal maintenance while still being dependent on its GEF function. Remarkably, our data also shows a previously unidentified interaction between AGEF-1 and RAB-35, where the two molecules are in proximity *in vivo*. In addition, we show that AGEF-1 functions upstream of active RAB-35. This raises the possibility that AGEF-1 could function as a GEF for RAB-35, or that it could bind RAB-35 and modulate its activity through an intermediate GTPase in a molecular complex. Indeed, the yeast ortholog of AGEF-1, Sec7, can function as an effector for Ypt1 (a homolog of mammalian Rab1) and Ypt31 and Ypt32 (homologs of mammalian Rab11 proteins) (McDonold and Fromme, 2014). Moreover, it has been shown that Arf5 allosterically activates DENND1 (ortholog of RME-4) GEF activity towards Rab35 in mice glioblastoma derived cells, upregulating clathrin-mediated endocytosis and leading to a down-modulation of tumor invasiveness (Kulasekaran et al., 2021). Interestingly, in previous work, it has been demonstrated that BIG2 expression induces a small increase in Arf5-GTP levels in HeLa cells (Shin et al., 2004). Here, we show that ARF-5, which is 80% identical to human Arf5 and a validated target GTPase of AGEF-1 (Skorobogata et al., 2014), functions in axonal maintenance possibly upstream of active RAB-35. Considering this result, together with our observations that AGEF-1 is in proximity to RAB-35 and functions redundantly with RME-4, it is possible that these molecules form a complex *in vivo*. Elucidating the physical interaction between these four molecules will expand our targets on how to modulate the activity of Rab35.

How do AGEF-1, RAB-35 and UNC-70 affect neuron–epidermal attachment? AGEF-1 is a known modulator of the basolateral polarity of the epidermal growth factor LET-23 and protein secretion (Skorobogata et al., 2014). Furthermore, it is implicated in the regulation of endosome-lysosome fusion and fission as well as endocytic transport (Tang et al., 2012). RAB-35 in both *C. elegans* and humans, as well as UNC-70 and β-spectrin, have also been described to be involved in different steps of endocytosis and membrane recycling, and their exact function in these processes seems complex (Coakley et al., 2026; Haley et al., 2018; Sato et al., 2008; Wernert et al., 2024; Xu et al., 2024). In line with these findings, our work supports the idea that UNC-70 stabilizes adhesive membrane domains, whereas RAB-35 and AGEF-1 activity further destabilize these attachments in the absence of UNC-70. These notions support the concept that membrane recycling events within the epidermal membrane closest to an innervating neurite are crucial for the maintenance of axonal integrity. Consequently, a trans-tissue coordination of membrane domain organization is required to support organismal health, especially between tissues that are under constant mechanical challenge.

## MATERIALS AND METHODS

### Strains

Nematode cultures were grown in nematode growth medium (NGM) plates seeded with OP50 *Escherichia coli* and maintained at 20°C following standard methods (Brenner, 1974). Animals were visually selected at the last larval stage on a stereomicroscope and then scored for PLM axon breaks 48 h later as 2-day-old adults. Mutant animals of genotype *tbc-10(vd31); vdSi2[Pdpy-7::UNC-70(n493)::mKate2]; zdIs5[Pmec-4::GFP]* were generated previously (Coakley et al., 2020). Strains used in this work are listed in Tables S1 and S2.

### Isolation of mutant strains

Animals of genotype *vdSi2[Pdpy-7::UNC-70(n493)::mKate2]; tbc-10(vd31); zdIs5* were mutagenized using 50 mM ethyl methanesulfonate (EMS, Sigma) for 4 h. *vd92* mutants were isolated in a clonal F2 progeny screen and then backcrossed three times with the original sensitized strain QH7929 [*tbc-10(vd31); vdSi2[Pdpy-7::unc-70(n493)::mKate2]* before whole-genome sequencing was conducted (GeneWiz). Strains QH8151 [*vd92; tbc-10(vd31); vdSi2[Pdpy-7::UNC-70(n493)::mKate2]; zdIs5[Pmec-4::GFP]*], QH7929 [*vdSi2[Pdpy-7::UNC-70(n493)::mKate2]; tbc-10(vd31); zdIs5[Pmec-4::GFP]*], CZ10175 [*zdIs5[Pmec-4::GFP]*] and the N2 Bristol strain were whole-genome sequenced at a mean depth of ≥19. Genome mapping and calling of variants were performed by GeneWiz. Background variants subtraction and candidate mutation filtering and mapping was performed using a customized Python script following a strategy of direct mapping (Zuryn et al., 2010).

### Genetic engineering

Knockins, single base pair changes and deletions were made with CRISPR-Cas9 using a co-CRISPR strategy (Kim et al., 2014). An equimolar mix of ALT-R Cas9, tracRNA and ALT-R crRNA (Integrated DNA Technologies) at a final concentration of 1.525 μM plus single-stranded oligonucleotides (Integrated DNA Technologies) repair templates at a final concentration of 1.6 μM, or PCR products in a concentration range of 0.3–0.5 μM, were diluted in ultrapure water and microinjected into the gonads of adult animals. All repair templates were designed with 75 base pair homology to flanking genomic regions. PCR repair templates were amplified using ultramer oligonucleotides or obtained as gBlocks (Integrated DNA Technologies). For deletions, no repair templates were used. Sequences of all crRNAs and oligonucleotides used are listed in Table S3. Final engineered lines were genotyped by PCR and Sanger sequencing.

### RNA interference

*arf-*5 and *warf-1* RNAi (clones F57H12.1 and F45E4.1, respectively) (Kamath and Ahringer, 2003) were induced by feeding (Timmons et al., 2001). 50 mg/ml ampicillin, 12.5 mg/ml tetracycline, and 1 mM isopropyl β-D-1-thiogalactopyranoside (Astral Scientific P/L) were added to standard NGM agar, poured into 35 mm petri dishes, and allowed to dry overnight at room temperature. *E. coli* HT115 carrying either the pL4440 control empty vector (pL4440; Addgene plasmid #1654, deposited by Andrew Fire) or the test RNAi-inducing clones, were grown in 2 ml of LB containing 50 mg/ml ampicillin and 12.5 mg/ml tetracycline at 37°C with shaking for 6 h. 100 μl of these cultures were then seeded onto the above supplemented NGM agar plates and allowed to dry and induce for 2 days at room temperature. Twenty 1-day-old adult animals were added to RNAi plates, allowed to lay eggs for 3 h at 20°C and then removed. Their synchronized F1 progeny was then scored 96 h later (approximately at the 2-day-old adult stage).

### Auxin-inducible degradation

NGM plates (60 mm) seeded with *E. coli* OP50 were soaked with 320 μl of 25 mM 1 mM 1-naphthaleneacetic acid potassium salt (K-NAA; GK2088; Glentham Life Sciences) diluted in milliQ water and allowed to dry overnight in a dark chamber. To induce degradation, animals from strain COA795 were transferred to 1 mM K-NAA plates at the L4 stage and scored 48 h later. For controls, siblings were raised on regular OP50 plates without K-NAA in the same conditions, selected at the L4 stage and scored 48 h later.

### Transgenics

Transgenic strains carrying extrachromosomal arrays were generated by microinjection into the germline using standard methods (Mello and Fire, 1995). All constructs were made using the Gibson isothermal assembly method (Gibson et al., 2009; Rabe and Cepko, 2020 preprint) except Pdpy-7::mKate::AGEF-1[608K], which was generated using the quick-change mutagenesis II kit (Agilent). DNA fragments for assembly were generated by PCR. pSM vectors (derivatives of Fire vector, developed in the laboratory of Cori Bargmann, Rockefeller University, New York, USA) were utilized as the backbone for assembly. The sequence of all constructs was validated by Sanger sequencing. The sequences of all constructs are listed in Table S2. AGEF-1 and BIG2 cDNA were obtained from Genescript based on the sequence of WormBaseID:Y6B3A.1a. AGEF-1[E608K] sequence was determined by protein alignment of AGEF-1 and BIG2[E738K] (Shinotsuka et al., 2002b) then generated using construct Pdpy-7::mKate::AGEF-1 as template.

### Microscopy

Animals were inspected by mounting on 4% agar pads after anesthesia with 0.05% tetramisole hydrochloride in M9 buffer (Stiernagle, 2006). Fluorescence microscopy was performed using an upright Zeiss AxioImager A1 microscope equipped with a Photometrics Cool Snap HQ2 camera. Images were acquired in Metamorph^®^. Confocal imaging was performed using a spinning disk confocal microscope (Marianas, Intelligent Imaging Innovations), equipped with a confocal scanner unit (CSU-W1, Yokogawa Electric Co.) built around an Axio Observer body (Z1, Carl Zeiss AG) and fitted with an sCMOS camera (ORCA-Flash4.0 V2, Hamamatsu Photonics) and SlideBook v6.0 software (3i) using a 63×/1.4 NA oil-immersion objective with sampling intervals *x*, *y*=99 nm and *z*=130 nm. Image acquisition was performed using SlideBook 6.0 (3I, Inc). Detector-array super resolution imaging was performed using a Zeiss C Plan-Apochromat 63×/1.4 NA oil-immersion objective on a confocal/two-photon laser-scanning microscope (LSM 980 NLO Airyscan 2, Carl Zeiss GmbH) built around an Axio Observer 7 body and equipped with an Airyscan 2 super-resolution detector, a 34-channel spectral photomultiplier tube (PMT) array, two internal GaAsP PMTs, a transmission PMT, and two external GaAsP PMTs for non-descanned detection in two-photon microscopy. Both confocal and detector-array imaging were done in the Queensland Brain Institute's Advanced Microscopy Facility. Confocal microscope acquisitions were deconvolved using Huygens Professional version Huygens Professional v18.04 (Scientific Volume Imaging, The Netherlands) run on a GPU-accelerated computer (3x NVIDIA^®^ Tesla^®^ V100), using the CMLE algorithm with SNR:20 and 32 iterations. Detector-array microscope acquisitions were deconvolved using the GMLE algorithm, with SNR:8.31, Acuity: 20 and 5 iterations. Microinjections were performed using standard methods (Mello and Fire, 1995), on either an inverted Zeiss AxioObserver microscope equipped with differential interference contrast, a Narishige needle holder, and a Tritech Research injection system.

### Image analysis and panel preparation

All images were analyzed in FIJI software (Schindelin et al., 2012). LET-805::wrmScarlet localization scoring was done using a custom FIJI macro (available upon request) to segment the analysis region with the aid of the plugin SNT (Arshadi et al., 2021) to trace the axon and then generate a line profile of the LET-805::wrmScarlet channel in that area. A custom Python script (Zenodo, doi:10.5281/zenodo.6031709) was made to analyze the first 100 μm of the PLM axon line profiles. Using this data as input, the program then scored animals with gaps in LET-805::wrmScarlet larger than ∼5 μm as ‘gaps’, animals with continuous LET-805::wrmScarlet as ‘continuous’ and animals with a PLM break as ‘gap and break’. A gap was defined as a bin of ∼5 μm in length where the sum of its signal is less than 21% of the maximum possible signal in such a bin. For quantification of GFP reconstitution and mScarlet3::RAB-35 expression, three non-overlapping regions of interest (ROIs) of 10 μm were randomly selected along the vicinity proximal to the PLM neuron axon anatomical location for analysis. Segmentation of GFP puncta was done through simple thresholding using the FIJI implementation of the Li threshold method while segmentation of mScarlet3::RAB-35 signal was done through the Otsu threshold method. mScarlet3::RAB-35 integrated density value was measured from each ROI and values were normalized by the average wild-type value. Automated quantification of segmented puncta was performed using the FIJI plugin Particle Analysis (parameters: size=0.04–3; circ=0.5–1; exclude on edge). Further filtering of quantified puncta was performed in Python to exclude puncta that had a perimeter and mean intensity smaller than the 25th percentile of such measurements of all puncta. Figure panels were prepared using FigureJ (Mutterer and Zinck, 2013) and Adobe Illustrator^®^.

### Protein sequence alignment and modeling

AGEF-1a amino acid sequence was aligned to its closest orthologs in *Danio rerio*, *Drosophila melanogaster, Mus musculus* and *Homo sapiens* using the alignment tool in https://www.uniprot.org/ (The UniProt Consortium, 2021). Respective UniProt IDs for each molecule were: G5EFH7_CAEEL, E7FCG1_DANRE, Q9VJW1_DROME, BIG2_MOUSE, BIG2_HUMAN. Uniprot IDs used for sequence alignment of nematode ARF-5 and human Arf5: ARF12_CAEEL, G5EFK4_CAEEL, ARF5_HUMAN. 3D model generated using AlphaFold 3 (Abramson et al., 2024) using G5EFH7_CAEEL and Q9U2C3_CAEEL, to model AGEF-1 and RAB-35, respectively. Model alignment and scene adjustment done using PyMol (https://pymol.org/).

### Statistical analysis

Statistical analysis and plotting were performed using GraphPad Prism version 11 for Windows (GraphPad Software, La Jolla California USA). One-way ANOVA followed by Tukey's multiple comparisons test was used to compare the mean of multiple groups. Unpaired, two-tailed *t-*tests were used to compare pairs.

## Supplementary Material



10.1242/joces.264565_sup1 Supplementary information

Table S1. Stable lines utilized in this study.

Table S2. Semi-stable transgenic lines utilized in this study.

Table S3. Oligonucleotides and crRNAs utilized in this study.
