# Supplementary material for: A conserved Arf-GEF modulates axonal integrity through RAB-35 by altering neuron–epidermal attachment
Source: J Cell Sci. 2026 Jul 13;139(13):jcs264565. doi: 10.1242/jcs.264565 (PMC13405217; doi:10.1242/jcs.264565)
Supplement: Supplementary information [file joces-139-264565-s1.pdf]

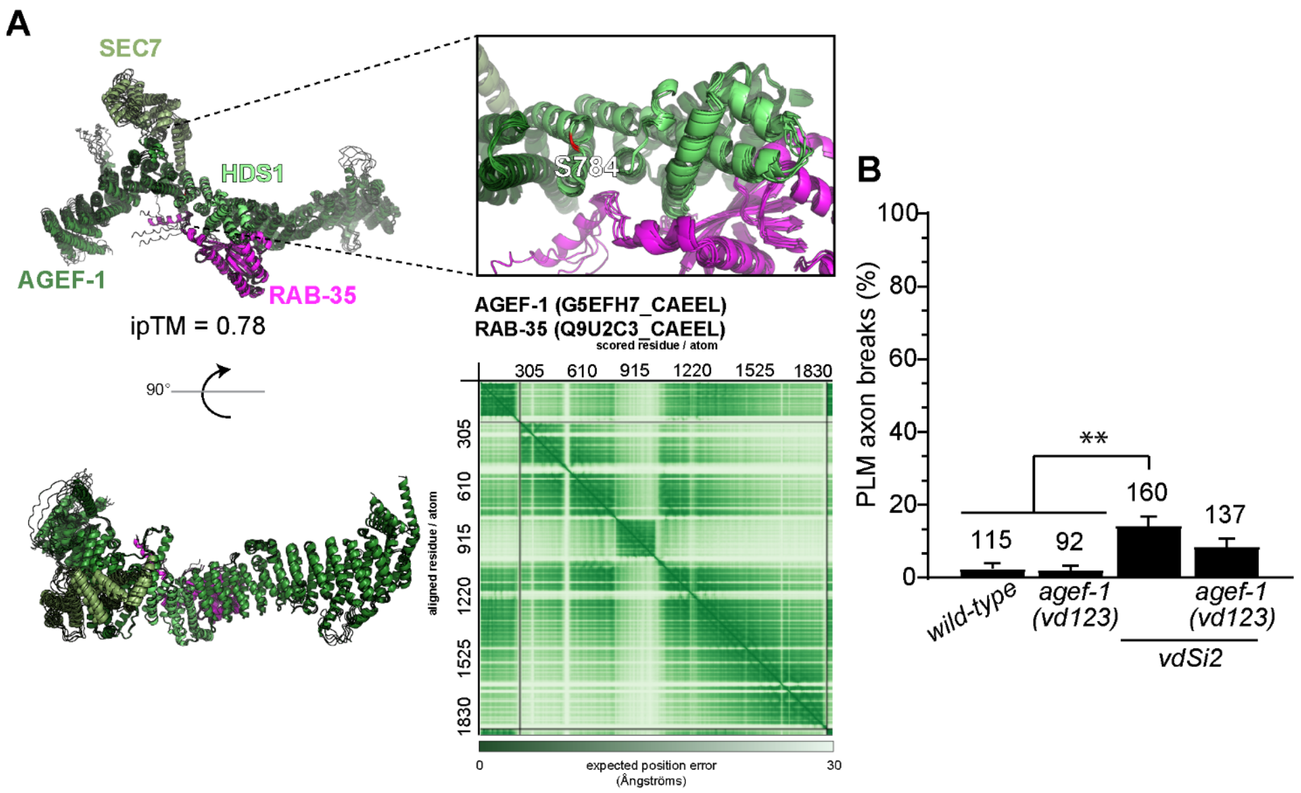

Fig. S1. S784L mutation falls in the HDS1 domain and does not cause axonal damage. (A) Alignment of the top 4 AlphaFold 3D structural models of the possible interaction between AGEF-1 (green) and RAB-35 (magenta) highlighting the location of the catalytic site (SEC7 domain), HDS1 domain. Enlarged box showing the position of Serine 784 relative to the predicted binding interface between AGEF-1 and RAB-35. Position alignment error plot of the predicted complex. (B) Mean penetrance of PLM axon breaks in *agef-1(vd123), vdSi2 and agef-1(vd123); vdSi2* animals. Bars represent the mean penetrance based on a uniform-prior distribution. Standard error of the mean and sample size are represented on top of the bars. ANOVA used to compare multiple groups. **; $p < 0.01$.

Journal of Cell Science • Supplementary information

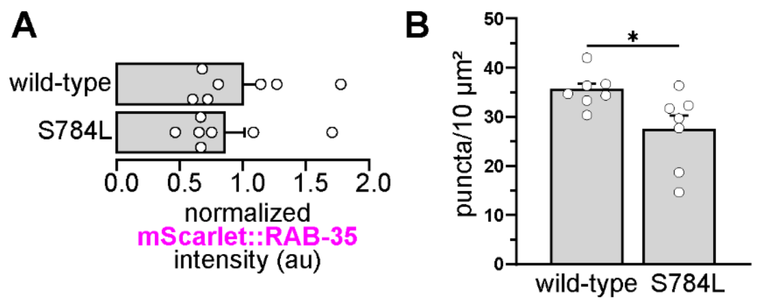

Fig. S2. Morphological parameters of mScarlet3::RAB-35, and GFP reconstitution strains. (A) Mean normalized intensity of mScarlet3::RAB-35 expression in AGEF- 1::GFP$_{11x7}$ and AGEF-1[S784L]::GFP$_{11x7}$ animals. Samples normalized by the mean intensity of the wild-type group. (B) Quantification of the average number of GFP positive puncta in engineered AGEF-1::GFP$_{11x7}$ and AGEF-1[S784L]::GFP$_{11x7}$ animals expressing cytosolic SKIN::GFP$_{1-10}$. White circles represent individual samples. Standard error is represented on top of bars. t-test used to compare pairs. *, $p<0.05$.

Journal of Cell Science • Supplementary information

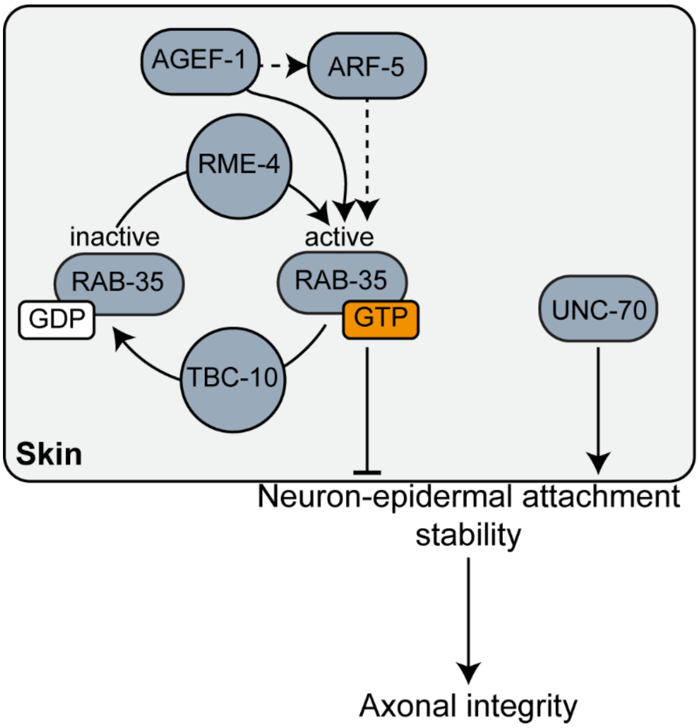

Fig. S3. AGEF-1 functions in redundance with RME-4 to activate RAB-35 in the epidermis and modulate neuron-epidermal attachment stability. Schematic of the genetic pathway in the skin regulating neuron-epidermal attachment stability, and axonal integrity. Arrows indicate a positive regulation, while capped line indicates a negative regulation. Solid lines indicate directly validated gene interactions; dashed lines indicate predicted genetic interactions.

Table S1. Stable lines utilized in this study.

Available for download at
https://journals.biologists.com/jcs/article-lookup/doi/10.1242/jcs.264565#supplementary-data

Table S2. Semi-stable transgenic lines utilized in this study.

Available for download at
https://journals.biologists.com/jcs/article-lookup/doi/10.1242/jcs.264565#supplementary-data

Table S3. Oligonucleotides and crRNAs utilized in this study.

Available for download at
https://journals.biologists.com/jcs/article-lookup/doi/10.1242/jcs.264565#supplementary-data

Journal of Cell Science • Supplementary information
