# Supplementary material for: A conserved Arf-GEF modulates axonal integrity through RAB-35 by altering neuron–epidermal attachment
Source: J Cell Sci. 2026 Jul 13;139(13):jcs264565. doi: 10.1242/jcs.264565 (PMC13405217; doi:10.1242/jcs.264565)
Supplement: Supplementary file 1 [file joces-139-264565_review_history.pdf]

A conserved Arf-GEF modulates axonal integrity through RAB-35 by altering neuron-epidermal attachment

Igor Bonacossa-Pereira, Dat Le, Sean Coakley and Massimo Hilliard
DOI: 10.1242/jcs.264565

Editor: Subhojit Roy

Review timeline

Submission to Review Commons:	8 June 2025
Submission to Journal of Cell Science:	5 November 2025
Editorial decision:	20 November 2025
First revision received:	14 April 2026
Editorial decision:	9 May 2026
Second revision received:	27 May 2026
Accepted:	5 June 2026

Reviewer 1

Evidence, reproducibility and clarity

Summary

Stability of the PLM axon in C. elegans is maintained through interactions with the epidermis. Previous studies by this group found that loss of the tbc-10 Rab GTPase Activating Protein strongly enhanced the PLM axon break phenotype of unc-70/beta-spectrin mutants. TBC-10 is a GAP for RAB-35 and thus loss of rab-35 suppresses the tbc-10 phenotype. Of the two RAB-35 GEFs, loss of RME-4 partially suppressed the tbc-10 phenotype and FLCN-1 was not involved suggesting that there may be an additional GEF involved. Here Bonacossa- Pereira et al identify a point mutation in agef-1a (vd92) as a suppressor of tbc-10 PLM axon break phenotype (all experiments also have a dominant allele of unc-70) and confirm that point mutation is causative by replicating the mutation via genome editing (vd123). Rescue experiments demonstrate that AGEF-1a is required in the epidermis and not PLM as previous demonstrated with tbc-10 and unc-70. Rescue is dependent on a functional SEC7/GEF activity. AGEF-1a is a functional ortholog to human BIG2/ArfGEF2 as its expression fully rescues tbc-10. AGEF-1a functions upstream of RAB-35 as expression of activated RAB-35 can suppress loss of agef-1. AGEF-1a functions in parallel to RME-4 as the double has stronger suppression of tbc-10. AGEF-1a is an ARF GEF, however it functions independently of ARF-1.2 as loss of arf-1.2 does not suppress tbc-10. They demonstrate that AGEF-1a interacts with RAB-35 through colocalization experiments suggesting that AGEF-1a could directly activate RAB-35. Finally, they demonstrate that AGEF-1a regulates the localization of the LET-805 epidermal attached complex component as it restores localization in a tbc-10 mutant.

Major comments

The manuscript is well written and easy to understand.
The experiments are well done and controlled.
I enjoyed reading this paper. However...

Some of the claims are not supported by the data.

© 2026. Published by The Company of Biologists under the terms of the Creative Commons Attribution License (https://creativecommons.org/licenses/by/4.0/).

1. The claim that AGEF-1a directly interacts with RAB-35 was not demonstrated. The evidence provided to support a direct interaction are colocalization experiments in Figure 3. AGEF-1a does partially colocalize with RAB-35 in the epidermis. However, colocalization does not indicate a physical interaction direct or indirect. A simple fix would be to change the claim to that they partially colocalize. Optional, a physical interaction could be done with the split-GFP since they already have the AGEF-1 strain or they could perform co-IP experiments, though neither of those are proof of direct interactions.
2. The claim that AGEF-1a facilitates RAB-35 activation is not supported. While it is likely that AGEF-1a facilitates RAB-35 activation based on the epistasis experiments as well as studies in mammalian cells there were no experiments to demonstrate that modulating AGEF-1a activity resulted in a change in RAB-35 activity. I would suggest tempering this claim to something along the line that the data are consistent with AGEF-1a regulating RAB-35 activity as shown in mammalian cells. An optional experiment would be to look at the colocalization of RAB-35 with a known effector in wild type and agef-1(vd92) with the expectation that there would be a higher level of colocalization in agef-1 mutants. Effector pull-down experiments or perhaps a cell based GEF assay could be used (PMID: 35196081).
3. The claim that AGEF-1a functions independently of ARF-1.2 is not well supported. The fact that the ARF-1.2 mutant does not suppress tbc-10 suggests that ARF-1.2 may not be involved but does not eliminate the possibility that ARF-1.2 functions redundantly with ARF-5 or WARF-1/ARF-1.1. This can be resolved by toning down the claim. Alternatively, this can be tested by RNAi of arf-5 and warf-1 in tbc-10 and arf-1.2; tbc-10 mutants.

Minor comments

Figure 1C the CRISPR generated allele (vd123) is referred to as [S784L] and then in 1E vd92 is referred to as [S784L]. Perhaps it would be clearer if the allele name was used instead of the amino acid change.

Page 6 "We reasoned that if the S784L mutation we isolated causes a similar loss of the GTPase activation function, then SKIN::AGEF-1a[E608K] would not have the capacity to restore the rate of PLM axon breaks to background levels in agef-1[S784L]; tbc-10; vdSi2 animals." It was unclear to me whether you were testing if the S784L mutation could be disrupting a GEF independent function or might disrupt the nucleotide exchange activity as might be tested in a biochemical assay. There are many reasons this change could cause a loss of function phenotype (ie. Improper folding, mislocalization, etc.). The most clear explanation would be that you were testing if GEF function was required for rescue rather than testing if the S784L mutation disrupted GEF activity.

Page 13. It was unclear how testing if AGEF-1, RME-4, ARF-5 and RAB-35 form complexes in vivo (I assume you are suggesting colocalize based on figure 3 interpretation) would resolve how AGEF-1 was regulating RAB-35.

Cross-commenting

I agree with the comments made by the other reviewers and I stand by my own as well. I will echo that it is important to know the nature of their agef-1 allele.

Significance

Bonacossa-Pereira et al identify AGEF-1 as a regulator of axon integrity that functions in a pathway with RAB-35 in the epidermis is an exciting finding. As pointed out in the discussion, mutations in the human ortholog cause neurodevelopmental defects which leads to obvious characterization of BIG2/ArfGEF2 in neurons while this study indicates that this protein can have cell non-autonomous roles in regulating neurons. These findings could have important implications for understanding the etiology of these defects that would be of interest to neurobiologists and clinical researchers.

The finding of this paper would also be of interest to cell biologists and particularly those studying the roles of Rab and Arf GTPases in membrane trafficking, such as myself. The idea that AGEF-1 might function as a Rab35 GEF is provocative and would generate a lot of interest and skepticism from the field. However, there is no data to support that AGEF-1 would be a direct regulator of

© 2026. Published by The Company of Biologists under the terms of the Creative Commons Attribution License (https://creativecommons.org/licenses/by/4.0/).

Rab35 over the previously demonstrated cross regulation of Rab35 by Arf GTPases. Therefore, it would be fine to speculate in the discussion a direct interaction, but I would refrain from suggesting this as a model and elsewhere in the manuscript.

Reviewer 2

Evidence, reproducibility and clarity

This interesting manuscript reports the outcome of a fruitful C. elegans genetic screen with a complex but clever design. Through it, the authors identify AGEF-1 as a GEF that likely regulates the active state of the GTPase RAB-35 in the skin to protect touch receptor axons from mechanical breakage.

Major points:

1. Based on localization experiments, the authors claim "AGEF-1a interacts with RAB-35 in the epidermis" (Results heading) and state "these data demonstrate that AGEF-1a interacts with a subset of RAB-35 molecules in the epidermis." In general, localization studies cannot be used to conclude physical interaction (with some exceptions such as single-molecule kinetics). In this case, the data in my view do not even make a compelling argument for co- localization. There is a lot of AGEF-1 and RAB-35 signal everywhere and it may not be meaningful that the signals sometimes overlap. A more quantitative approach with controls would be needed to conclude meaningful co-localization. Importantly, this would still not demonstrate interaction.
2. The effect of the AGEF-1(S784L) mutation is not clear to me. Naively, as the S784L mutation lies in the auto-inhibitory domain, I would have expected AGEF-1 to become constitutively active, not inactive as the authors seem to suggest. Is the idea that it is constitutively auto-inhibited? The main evidence for a loss of function effect seems to be that a putative dominant negative mutation AGEF-1(E608K) does not further supress axon breakage when co-expressed in trans to AGEF(S784L), but in my view this only shows that, once the defect is suppressed, it cannot be suppressed any further. Defining the nature of the S784L allele is important. Some suggestions, although the authors may come up with different approaches: use of an inducible or cell-specific depletion system like AID/TIR1, Cre/lox, or FLP/FRT to circumvent the lethality of agef-1(0) and reveal what a true loss-of- function looks like; testing if deletion of the auto-inhibitory domain phenocopies S784L to test if this mutation impairs autoinhibition.

Minor points:

1. I am not able to see the "vesicle-like structures with a clear luminal space" or RAB-35 being "notably enriched at the membrane near the epidermal furrow" in Fig. 3. The "3D surface rendering" in Fig. 3e is grossly oversampled and should not be included.
2. As the agef-1a isoform is specifically referenced throughout, please describe the different agef-1 isoforms somewhere to save readers from having to look this up.
3. The authors include an interesting speculation in the Discussion: "Future investigations of BIG2-associated neurological disorders should consider... hyper-activity of BIG2 as a driver of neuropathology." If the authors have the tools to test the effect of hyperactive BIG2 in this system, it could be an exciting addition.
4. On a personal note, since GEFs act oppositely to GTPase Activating Proteins (GAPs), I had to stop and re-read carefully whenever the authors referred to a GEF "activating" a GTPase. I understand their meaning (i.e., putting the GTPase in its active GTP-bound state, not activating its GTPase function) but I wanted to point out this potential confusion in case there is a way to better define terms in the Introduction or change word choice. I realize this may be a standard jargon in the field.
5. Please check the correct nomenclature for CRISPR/Cas9.
6. p.7 "these molecules act in synergy", consider replacing with "redundantly".

Significance

The significance of this story is to show that GEF-GTPases pairing can be highly context-dependent. Previous studies have identified GEFs that pair with RAB-35 and GTPases that pair with AGEF-1, but the authors find that these factors have at best a modest role in the context of

© 2026. Published by The Company of Biologists under the terms of the Creative Commons Attribution License (https://creativecommons.org/licenses/by/4.0/).

skin-axon interactions. Instead, the authors suggest a novel GTPase-GEF pairing of RAB-35 with AGEF-1 and provide evidence that this relationship is conserved in the human homolog of AGEF-1. These results suggest that GTPase-GEF pairings depend not only on chemical affinity but also cellular context.

The main strength of the study is its clever genetics. For the screen, the authors looked for suppressors of a synthetic defect in axon integrity caused in part by elevated activity of RAB-35 due to loss of its GAP TBC-10. It is satisfying that this screen isolated a mutation in a GEF that in principle could counterbalance the loss of a GAP.

The main weakness of the study is the lack of direct evidence for an AGEF-1/RAB-35 interaction. While not necessary for publication, the inclusion of biochemical data to support the role of AGEF-1 as a GEF for RAB-35 and the effect of the S784L mutation on this activity would strongly elevate the study. The genetic data for this interaction are consistent with the model but not conclusive, and in my view the colocalization data are not compelling. Nevertheless this is a solid genetic story with a clever screen.

Reviewer 3

Evidence, reproducibility and clarity

This paper investigates the mechanism by which molecular pathways in the skin protect the processes of nerves that innervate them from damage. The authors previously showed that spectrin and the small GTPase RAB-35 act in the epidermis of C. elegans to protect mechanosensory axons from breaking. In this paper they used a suppression screen to identify another gene involved in this process, an ARF-GEF called AGEF-1. Partial loss-of- function mutations in agef-1 suppress the axon-breakage phenotype of spectrin mutations, and genetic experiments by the authors are consistent with the possibility that AGEF-1 could act directly as an exchange factor for RAB-35. Consistent with this model, they show that AGEF-1 and RAB-35 colocalise in the skin.

Major comments: The experiments in this paper are well-designed and well-controlled, and the interpretations of the results are all reasonable. On the other hand, I don't think the authors' hypothesis that AGEF-1 acts directly as an exchange factor for RAB-35, or that these two proteins directly interact, is definitively proven. This is not an issue of the authors overinterpreting their data--the paper is very carefully and thoughtfully written. However, the most interesting and counterintuitive finding--that an ARF-GEF could also be a RAB-GEF-- might be strengthened with more experiments (for example, could they more directly show protein-protein interaction through co-IP or mass spec?).

Minor comments: There are also two places where the fact that null mutations are lethal (for agef-1 and arf-5) prevented the authors from addressing the effect of agef-1 loss of function in the skin, and addressing whether ARF-5 could be an AGEF-1 target, respectively. In principle, they could have tried to make a CRISPR line in which these genes could be cell- specifically deleted in the skin (using a dpy-7-driven recombinase). I don't think either of these experiments are essential, but if it is feasible to make these lines it would tie up a couple of loose ends.

Significance

Overall I think this is an interesting paper on a topic of general interest. The most interesting finding is that an exchange factor for an ARF (a small GRPase involved in vesicle coating/uncoating) could also be an exchange factor for a RAB (a small GTPase involved in vesicle tethering). The evidence presented is suggestive and intriguing, though as noted above not completely definitive. In summary, I think it is an interesting paper in its current form, and anything it could do to more firmly establish a direct interaction between AGEF-1 and RAB-35 would increase its impact and importance.

Reviewer 4

Evidence, reproducibility and clarity

© 2026. Published by The Company of Biologists under the terms of the Creative Commons Attribution License (https://creativecommons.org/licenses/by/4.0/).

Summary:

In this study Bonacossa-Pereira et al. identify AGEF-1a, an Arf-GEF, as a factor that functions in the epidermis through RAB-35 to regulate axonal integrity of the PLM mechanosensory neurons in C. elegans. Specifically, epidermal attachment sites are regulated by these genes form the epidermis and compromising these attachment sites results in axonal degeneration. The study provides some evidence that that RAB-35 and AGEF-1 at least partially colocalize in the skin. Finally, the authors provide evidence that the human orthologue BIG2 is capable of functionally replacing AGEF-1a in C. elegans. Overall, the experiments are well designed and the paper is clear and succinct. The conclusions are supported by the findings and provide an important extension of the author's findings a few back, when they identified the role of rab-35 in mediating the epidermal-neuronal attachment sites.

Major comments:

1. AGEF-1/BIG2 are known to regulate other GTPases such as ARF-5 or ARF-2. The authors exclude a non-redundant function for ARF-2, but are unable to establish a role for ARF-5 because of the lethality associated with the mutation. Alternative approaches, such as cell specific knock out or knock down experiment. In addition, studies to test potentially physical interaction such as pull-down assays, co-IP experiments and FRET could be used to test whether AGEF-can bind RAB-35 or ARF-5.
2. Phenotypic readout has been limited to only axon breaks. It may be interesting to also test other aspects such as axonal deformities including swellings and vesiculation in other parts of the nervous system. Moreover, behavioral or functional experiments such as response to gentle touch orsynaptic integrity could be informative.
3. Overexpression constructs such as SKIN::RAB-35[Q69L], SKIN::BIG2, SKIN::AGEF-1a[E608K] in extrachromosomal transgenes could lead to non-physiological localization or effects. Single copy expression using MosSCI or CRISPR insertions are generally considered better approaches (other than endogenous reporters) to provide accurate

insights at the physiological level. While the authors tacitly acknowledge this by conducting the experiments in a rab-35 mutant background and very low transgene concentration, at the very least this caveat regarding the localization should be discussed.
4. The study does not address clearly whether AGEF-1a acts in parallel to spectrin or upstream/ downstream to it. Epistasis experiments could help to figure out the signaling pathway involved.
5. The finding that BIG2 rescues the mutant defect is an important finding and rightfully finds its place in the abstract. I wonder whether a reference to the human diseases caused by loss of BIG2 in the abstract and introduction would not increase interest/impact for the study, rather than burying this potentially interesting connection in the discussion.

Minor comments:

1. Some explanation about how mutating the autoinhibitory domain could impact the catalytic activity of a GEF might be helpful.
2. The paper refers to rme-4(b1001) as a null allele while wormbase refers to the same as a missense allele. It would be more accurate to refer rme-4(b1001) as a strong loss of function or putative null.
3. The paper does not clearly discuss limitations of the hypomorphic agef-1[S784L] and that the observed phenotypes in this hypomorph might underestimate the complete role of AGEF-1a.
4. In figure 1, where there really only one extrachromosomal transgenic line for some of the construct tested?
5. The concentrations of transgenes vary in different transgenes. Is there a rationale behind this?
6. In Fig.1e: I may be useful to also show the "WT" phenotype, i.e. the strong defects to get a visual comparison for the degree of rescue.

Significance

© 2026. Published by The Company of Biologists under the terms of the Creative Commons Attribution License (https://creativecommons.org/licenses/by/4.0/).

The study has identified AGEF-1a as a regulator of axonal maintenance, functioning to protect neurons against mechanical stress by acting through RAB-35. Additionally, this epidermal GEF, AGEF-1a is functionally conserved as its human orthologue BIG2 can replace AGEF-1a in C. elegans for axonal protection. Important points here are that the findings extend prior work by the authors of non-autonomous mechanism that regulates epidermal-neuronal attachment. In my humble opinion, the human disease connection, in particular with regard to the unexplained neuronal phenotypes in patients could be better developed in the manuscript. It may also increase impact/interest of a wonderful story that right now reads a bit 'wormy'

Original submission

First decision letter

MS ID#: jcs.264565

MS TITLE: A conserved Arf-GEF modulates axonal integrity through RAB-35 by altering neuron-epidermal attachment

AUTHORS: Igor Bonacossa-Pereira; Sean Coakley; Massimo Hilliard

ARTICLE TYPE: Review Commons Transfer

Dear Dr Hilliard,

Many thanks for transferring your paper to Journal of Cell Science from Review Commons. Following initial assessment, we have sought further input from the Review Commons referees. I have now received their feedback and have reached a decision.

I agree with the detailed revision plan. The additional experiments to determine interactions between AGEF-1 and Rab-35 are particularly important. One consideration might be to stain for an unrelated Rab to determine what a "random localization" might look like. Quantification of this colocalization is also important.

Please attend to all of the reviewers' comments and ensure that you clearly highlight all changes made in the revised manuscript. Please avoid using 'Tracked changes' in Word files as these are lost in PDF conversion. I should be grateful if you would also provide a point-by-point response detailing how you have dealt with the points raised by the reviewers in the 'Response to Reviewers' box. If you do not agree with any of their criticisms or suggestions please explain clearly why this is so.

Author response to reviewers' comments

Reviewer #1(Evidence, reproducibility and clarity (Required)):

Summary

Stability of the PLM axon in C. elegans is maintained through interactions with the epidermis. Previous studies by this group found that loss of the tbc-10 Rab GTPase Activating Protein strongly enhanced the PLM axon break phenotype of unc-70/beta-spectrin mutants. TBC-10 is a GAP for RAB-35 and thus loss of rab-35 suppresses the tbc-10 phenotype. Of the two RAB-35 GEFs, loss of RME-4 partially suppressed the tbc-10 phenotype and FLCN-1 was not involved suggesting that there may be an additional GEF involved. Here Bonacossa-Pereira et al identify a point mutation in agef-1a (vd92) as a suppressor of tbc-10 PLM axon break phenotype (all experiments also have a dominant allele of unc-70) and confirm that point mutation is causative by replicating the mutation via genome editing (vd123). Rescue experiments

© 2026. Published by The Company of Biologists under the terms of the Creative Commons Attribution License (https://creativecommons.org/licenses/by/4.0/).

demonstrate that AGEF-1a is required in the epidermis and not PLM as previous demonstrated with tbc-10 and unc-70. Rescue is dependent on a functional SEC7/GEF activity. AGEF-1a is a functional ortholog to human BIG2/ArfGEF2 as its expression fully rescues tbc-10. AGEF-1a functions upstream of RAB-35 as expression of activated RAB-35 can suppress loss of agef-1. AGEF-1a functions in parallel to RME-4 as the double has stronger suppression of tbc-10. AGEF-1a is an ARF GEF, however it functions independently of ARF-1.2 as loss of arf-1.2 does not suppress tbc-10. They demonstrate that AGEF-1a interacts with RAB-35 through colocalization experiments suggesting that AGEF-1a could directly activate RAB-35. Finally, they demonstrate that AGEF-1a regulates the localization of the LET-805 epidermal attached complex component as it restores localization in a tbc-10 mutant.

Major comments

The manuscript is well written and easy to understand.

The experiments are well done and controlled.

I enjoyed reading this paper. However...

Some of the claims are not supported by the data.
1) The claim that AGEF-1a directly interacts with RAB-35 was not demonstrated. The evidence provided to support a direct interaction are colocalization experiments in Figure 3. AGEF-1a does partially colocalize with RAB-35 in the epidermis. However, colocalization does not indicate a physical interaction direct or indirect. A simple fix would be to change the claim to that they partially colocalize. Optional, a physical interaction could be done with the split-GFP since they already have the AGEF-1 strain or they could perform co-IP experiments, though neither of those are proof of direct interactions.

We thank the Reviewer for suggesting these experiments. Indeed, we probed the physical interaction between endogenous AGEF-1 and RAB-35 using a split-GFP assay in vivo. Our data demonstrates that these molecules are close enough to reconstitute GFP, supporting our initial claim that they interact directly by binding to each other. The new data are now ***in Figure 3E, F with results added to the main text on lines 239 to 258****.*

2) The claim that AGEF-1a facilitates RAB-35 activation is not supported. While it is likely that AGEF-1a facilitates RAB-35 activation based on the epistasis experiments as well as studies in mammalian cells there were no experiments to demonstrate that modulating AGEF-1a activity resulted in a change in RAB-35 activity. I would suggest tempering this claim to something along the line that the data are consistent with AGEF-1a regulating RAB-35 activity as shown in mammalian cells. An optional experiment would be to look at the colocalization of RAB-35 with a known effector in wild type and agef-1(vd92) with the expectation that there would be a higher level of colocalization in agef-1 mutants. Effector pull-down experiments or perhaps a cell based GEF assay could be used (PMID: 35196081).

We agree and have updated the abstract and changed the phrase "…interaction between AGEF-1a and epidermal RAB-35 to facilitate its activation" to "AGEF-1 binds to epidermal RAB-35 and regulates its activity" (see ***lines 29 to 30****). We also changed the main text to remove the phrase "…AGEF-1a facilitates RAB-35 activation" and restricted our claims to propose that AGEF-1 functions upstream of active RAB-35 to regulate its activity, as suggested. Please see* ***lines 101 to 102, 212 to 214, line 268 and lines 291-294****.*

3) The claim that AGEF-1a functions independently of ARF-1.2 is not well supported. The fact that the ARF-1.2 mutant does not suppress tbc-10 suggests that ARF-1.2 may not be involved but does not eliminate the possibility that ARF-1.2 functions redundantly with ARF-5 or WARF-1/ARF-1.1. This can be resolved by toning down the claim. Alternatively, this can be tested by RNAi of arf-5 and warf-1 in tbc-10 and arf-1.2; tbc-10 mutants.

We have followed the suggestion and tested the involvement of arf-1 and warf-1 through RNAi. Indeed, our data shows that warf-1 is not involved but that arf-5 is functioning to modulate axonal integrity likely upstream of active rab-35. We have incorporated the new results in ***Figure 2C, D***

© 2026. Published by The Company of Biologists under the terms of the Creative Commons Attribution License (https://creativecommons.org/licenses/by/4.0/).

*and have rewritten the section accordingly "Expanding on this concept, given that AGEF-1a/BIG2 is known to activate ARF-1..." in lines **202 to 211**.*

Minor comments
Figure 1C the CRISPR generated allele (vd123) is referred to as [S784L] and then in 1E vd92 is referred to as [S784L]. Perhaps it would be clearer if the allele name was used instead of the amino acid change.

We have reformatted the manuscript to reference the allele names instead of the amino acid change.

Page 6 "We reasoned that if the S784L mutation we isolated causes a similar loss of the GTPase activation function, then SKIN::AGEF-1a[E608K] would not have the capacity to restore the rate of PLM axon breaks to background levels in agef-1[S784L]; tbc-10; vdSi2 animals." It was unclear to me whether you were testing if the S784L mutation could be disrupting a GEF independent function or might disrupt the nucleotide exchange activity as might be tested in a biochemical assay. There are many reasons this change could cause a loss of function phenotype (ie. Improper folding, mislocalization, etc.). The most clear explanation would be that you were testing if GEF function was required for rescue rather than testing if the S784L mutation disrupted GEF activity.

*We have rewritten the proposed section to reflect the suggestion. Please see **lines 154 to 172**.*

Page 13. It was unclear how testing if AGEF-1, RME-4, ARF-5 and RAB-35 form complexes in vivo (I assume you are suggesting colocalize based on figure 3 interpretation) would resolve how AGEF-1 was regulating RAB-35.

*We have rewritten this section to express this concept more clearly. See lines **313 to 328**.*

****Cross-commenting****

I agree with the comments made by the other reviewers and I stand by my own as well. I will echo that it is important to know the nature of their agef-1 allele.

Reviewer #1 (Significance (Required)):

Bonacossa-Pereira et al identify AGEF-1 as a regulator of axon integrity that functions in a pathway with RAB-35 in the epidermis is an exciting finding. As pointed out in the discussion, mutations in the human ortholog cause neurodevelopmental defects which leads to obvious characterization of BIG2/ArfGEF2 in neurons while this study indicates that this protein can have cell non-autonomous roles in regulating neurons. These findings could have important implications for understanding the etiology of these defects that would be of interest to neurobiologists and clinical researchers.

The finding of this paper would also be of interest to cell biologists and particularly those studying the roles of Rab and Arf GTPases in membrane trafficking, such as myself. The idea that AGEF-1 might function as a Rab35 GEF is provocative and would generate a lot of interest and skepticism from the field. However, there is no data to support that AGEF-1 would be a direct regulator of Rab35 over the previously demonstrated cross regulation of Rab35 by Arf GTPases. Therefore, it would be fine to speculate in the discussion a direct interaction, but I would refrain from suggesting this as a model and elsewhere in the manuscript.

The revisions provided a better understanding of how AGEF-1 functions with RAB-35. While we do not have conclusive evidence that AGEF-1 functions as a GEF for RAB-35, our data demonstrate that AGEF-1 binds RAB-35 and modulates its activity. Importantly, we show that ablating this interaction has a direct effect on axonal integrity maintenance. The precise nature of how AGEF-1 acts on RAB-35 activity remains to be discovered.

Reviewer #2 (Evidence, reproducibility and clarity (Required)):

© 2026. Published by The Company of Biologists under the terms of the Creative Commons Attribution License (https://creativecommons.org/licenses/by/4.0/).

This interesting manuscript reports the outcome of a fruitful C. elegans genetic screen with a complex but clever design. Through it, the authors identify AGEF-1 as a GEF that likely regulates the active state of the GTPase RAB-35 in the skin to protect touch receptor axons from mechanical breakage.

Major points:
1. Based on localization experiments, the authors claim "AGEF-1a interacts with RAB-35 in the epidermis" (Results heading) and state "these data demonstrate that AGEF-1a interacts with a subset of RAB-35 molecules in the epidermis." In general, localization studies cannot be used to conclude physical interaction (with some exceptions such as single-molecule kinetics). In this case, the data in my view do not even make a compelling argument for co-localization. There is a lot of AGEF-1 and RAB-35 signal everywhere and it may not be meaningful that the signals sometimes overlap. A more quantitative approach with controls would be needed to conclude meaningful co-localization. Importantly, this would still not demonstrate interaction.

*We have performed further experiments as suggested by Reviewer 1 and addressed this aspect. Please see **Reviewer 1, point one for relevant changes**.*

2. The effect of the AGEF-1(S784L) mutation is not clear to me. Naively, as the S784L mutation lies in the auto-inhibitory domain, I would have expected AGEF-1 to become constitutively active, not inactive as the authors seem to suggest. Is the idea that it is constitutively auto-inhibited? The main evidence for a loss of function effect seems to be that a putative dominant negative mutation AGEF-1(E608K) does not further supress axon breakage when co-expressed in trans to AGEF(S784L), but in my view this only shows that, once the defect is suppressed, it cannot be suppressed any further. Defining the nature of the S784L allele is important. Some suggestions, although the authors may come up with different approaches: use of an inducible or cell-specific depletion system like AID/TIR1, Cre/lox, or FLP/FRT to circumvent the lethality of agef-1(0) and reveal what a true loss-of-function looks like; testing if deletion of the auto-inhibitory domain phenocopies S784L to test if this mutation impairs autoinhibition.

*We agree and have conducted further experiments to define the nature of our agef-1 allele by selectively degrading it in the skin of vdSi2; tbc-10 sensitized animals. Together with our previous data, our results reveal that the S784L mutation causes a partial loss-of-function that ultimately impairs GEF function, supporting a model where this mutation might enhance auto-inhibition. Please see **Figure 1G** and **Figure S1B** and **lines 166 to 172**.*

*We have rephrased our results to express the concept of how we believe the S784L mutation might affect auto-inhibition. See **line 154**.*

Minor points:
1. I am not able to see the "vesicle-like structures with a clear luminal space" or RAB-35 being "notably enriched at the membrane near the epidermal furrow" in Fig. 3. The "3D surface rendering" in Fig. 3e is grossly oversampled and should not be included.

*We have rephrased our description of the RAB-35 localization appearance in **lines 231 to 235** and acquired new confocal and super-resolution images to address these important concerns (**see Figure 3B-D**).*

2. As the agef-1a isoform is specifically referenced throughout, please describe the different agef-1 isoforms somewhere to save readers from having to look this up.

*We have included a brief explanation of the different isoforms in **lines 117 to 121** and redesigned the schematic in Figure 1 (**see Figure 1D**).*

3. The authors include an interesting speculation in the Discussion: "Future investigations of BIG2-associated neurological disorders should consider... hyper-activity of BIG2 as a driver of neuropathology." If the authors have the tools to test the effect of hyperactive BIG2 in this system, it could be an exciting addition.

© 2026. Published by The Company of Biologists under the terms of the Creative Commons Attribution License (https://creativecommons.org/licenses/by/4.0/).

This is an exciting idea, and one that we would like to keep in the Discussion. However, the biology of BIG2 activity regulation is a nascent field of research, and we believe that to accurately generate and characterise a hyperactive BIG2 would be beyond the scope of this manuscript.

4. On a personal note, since GEFs act oppositely to GTPase Activating Proteins (GAPs), I had to stop and re-read carefully whenever the authors referred to a GEF "activating" a GTPase. I understand their meaning (i.e., putting the GTPase in its active GTP-bound state, not activating its GTPase function) but I wanted to point out this potential confusion in case there is a way to better define terms in the Introduction or change word choice. I realize this may be a standard jargon in the field.

*We agree that this is a confusing nomenclature, and we have included a simpler explanation of their function in the introduction on **lines 79 to 83**.*

5. Please check the correct nomenclature for CRISPR/Cas9.

We have revised the text to use the name CRISPR-Cas9 consistently.

6. p.7 "these molecules act in synergy", consider replacing with "redundantly".

*We have revised as suggested in **lines 201**, **213**, **326**.*

Reviewer #2 (Significance (Required)):

The significance of this story is to show that GEF-GTPases pairing can be highly context-dependent. Previous studies have identified GEFs that pair with RAB-35 and GTPases that pair with AGEF-1, but the authors find that these factors have at best a modest role in the context of skin-axon interactions. Instead, the authors suggest a novel GTPase-GEF pairing of RAB-35 with AGEF-1 and provide evidence that this relationship is conserved in the human homolog of AGEF-1. These results suggest that GTPase-GEF pairings depend not only on chemical affinity but also cellular context.

The main strength of the study is its clever genetics. For the screen, the authors looked for suppressors of a synthetic defect in axon integrity caused in part by elevated activity of RAB-35 due to loss of its GAP TBC-10. It is satisfying that this screen isolated a mutation in a GEF that in principle could counterbalance the loss of a GAP.

The main weakness of the study is the lack of direct evidence for an AGEF-1/RAB-35 interaction. While not necessary for publication, the inclusion of biochemical data to support the role of AGEF-1 as a GEF for RAB-35 and the effect of the S784L mutation on this activity would strongly elevate the study. The genetic data for this interaction are consistent with the model but not conclusive, and in my view the colocalization data are not compelling. Nevertheless this is a solid genetic story with a clever screen.

*Please **see our reply to Reviewer 1 significance statement**.*

Reviewer #3 (Evidence, reproducibility and clarity (Required)):

This paper investigates the mechanism by which molecular pathways in the skin protect the processes of nerves that innervate them from damage. The authors previously showed that spectrin and the small GTPase RAB-35 act in the epidermis of C. elegans to protect mechanosensory axons from breaking. In this paper they used a suppression screen to identify another gene involved in this process, an ARF-GEF called AGEF-1. Partial loss-of-function mutations in agef-1 suppress the axon-breakage phenotype of spectrin mutations, and genetic experiments by the authors are consistent with the possibility that AGEF-1 could act directly as an exchange factor for RAB-35. Consistent with this model, they show that AGEF-1 and RAB-35 colocalise in the skin.

Major comments: The experiments in this paper are well-designed and well-controlled, and the interpretations of the results are all reasonable. On the other hand, I don't think the authors'

© 2026. Published by The Company of Biologists under the terms of the Creative Commons Attribution License (https://creativecommons.org/licenses/by/4.0/).

hypothesis that AGEF-1 acts directly as an exchange factor for RAB-35, or that these two proteins directly interact, is definitively proven. This is not an issue of the authors overinterpreting their data--the paper is very carefully and thoughtfully written. However, the most interesting and counterintuitive finding--that an ARF-GEF could also be a RAB-GEF--might be strengthened with more experiments (for example, could they more directly show protein-protein interaction through co-IP or mass spec?).

*We agree and have addressed this point, please **see Reviewer 1, point 1** for details.*

Minor comments: There are also two places where the fact that null mutations are lethal (for agef-1 and arf-5) prevented the authors from addressing the effect of agef-1 loss of function in the skin, and addressing whether ARF-5 could be an AGEF-1 target, respectively. In principle, they could have tried to make a CRISPR line in which these genes could be cell-specifically deleted in the skin (using a dpy-7-driven recombinase). I don't think either of these experiments are essential, but if it is feasible to make these lines it would tie up a couple of loose ends.

*We have addressed these concerns. Please **see Reviewer 2, major point 2 reply for more details**. For arf-5, please see our response to **reviewer 1, major point 3 for more details**.*

Reviewer #3 (Significance (Required)):

Overall I think this is an interesting paper on a topic of general interest. The most interesting finding is that an exchange factor for an ARF (a small GRPase involved in vesicle coating/uncoating) could also be an exchange factor for a RAB (a small GTPase involved in vesicle tethering). The evidence presented is suggestive and intriguing, though as noted above not completely definitive. In summary, I think it is an interesting paper in its current form, and anything it could do to more firmly establish a direct interaction between AGEF-1 and RAB-35 would increase its impact and importance.

*Please **see our reply to Reviewer 1 significance statement**.*

Reviewer #4 (Evidence, reproducibility and clarity (Required)):

Summary:
In this study Bonacossa-Pereira et al. identify AGEF-1a, an Arf-GEF, as a factor that functions in the epidermis through RAB-35 to regulate axonal integrity of the PLM mechanosensory neurons in C. elegans. Specifically, epidermal attachment sites are regulated by these genes form the epidermis and compromising these attachment sites results in axonal degeneration. The study provides some evidence that that RAB-35 and AGEF-1 at least partially colocalize in the skin. Finally, the authors provide evidence that the human orthologue BIG2 is capable of functionally replacing AGEF-1a in C. elegans. Overall, the experiments are well designed and the paper is clear and succinct. The conclusions are supported by the findings and provide an important extension of the author's findings a few back, when they identified the role of rab-35 in mediating the epidermal-neuronal attachment sites.

Major comments:
1. AGEF-1/BIG2 are known to regulate other GTPases such as ARF-5 or ARF-2. The authors exclude a non-redundant function for ARF-2, but are unable to establish a role for ARF-5 because of the lethality associated with the mutation. Alternative approaches, such as cell specific knock out or knock down experiment. In addition, studies to test potentially physical interaction such as pull-down assays, co-IP experiments and FRET could be used to test whether AGEF-can bind RAB-35 or ARF-5.

*We have addressed these concerns. Please see our response to **Reviewer 2, major point 2 for more details**. For data on other GTPases, please see our response to **Reviewer 1, major point 3 for more details**.*

2. Phenotypic readout has been limited to only axon breaks. It may be interesting to also test other aspects such as axonal deformities including swellings and vesiculation in other parts of

© 2026. Published by The Company of Biologists under the terms of the Creative Commons Attribution License (https://creativecommons.org/licenses/by/4.0/).

the nervous system. Moreover, behavioral or functional experiments such as response to gentle touch or synaptic integrity could be informative.

We agree that these are interesting ideas that would answer how the function of these, or other neurons, might be affected. However, we hope the Reviewers agree that expanding the study will be beyond out of the scope of this manuscript.

3. Overexpression constructs such as SKIN::RAB-35[Q69L], SKIN::BIG2, SKIN::AGEF-1a[E608K] in extrachromosomal transgenes could lead to non-physiological localization or effects. Single copy expression using MosSCI or CRISPR insertions are generally considered better approaches (other than endogenous reporters) to provide accurate insights at the physiological level. While the authors tacitly acknowledge this by conducting the experiments in a rab-35 mutant background and very low transgene concentration, at the very least this caveat regarding the localization should be discussed.

*We agree, and have addressed the RAB-35 localization concern by using CRISPR-Cas9 to overcome the possible artifacts caused by extrachromosomal array driven expression. We have included the new results in **Figure 3 B-D** and relevant textual changes in **lines 218 to 239**.*

For the cell-specific rescues, or dominant-negative constructs expression, we think that using extrachromosomal arrays is sufficient, since this allows us to compare genetically identical transgenic vs non-transgenic siblings with independent lines. Moreover, given these constructs are already driven by a tissue-specific promoter that is inherently stronger than their respective endogenous promoters, we believe that even a single-copy insertion would have the same caveats.

4. The study does not address clearly whether AGEF-1a acts in parallel to spectrin or upstream/ downstream to it. Epistasis experiments could help to figure out the signaling pathway involved.

*We have added a clearer explanation in **line 293** pointing to a new schematic in **Figure S3**, and included additional data on the genetic interaction between these molecules in **Figure S1B** with accompanying relevant text additions in **lines 128 to 130**.*

5. The finding that BIG2 rescues the mutant defect is an important finding and rightfully finds its place in the abstract. I wonder whether a reference to the human diseases caused by loss of BIG2 in the abstract and introduction would not increase interest/impact for the study, rather than burying this potentially interesting connection in the discussion.

*We have revised the abstract to include this suggestion (see **line 27**) and added it to the summary statement section on **lines 15 to 16**.*

Minor comments:

1. Some explanation about how mutating the autoinhibitory domain could impact the catalytic activity of a GEF might be helpful.

*Please see **Reviewer 2, major point 2**.*

2. The paper refers to rme-4(b1001) as a null allele while wormbase refers to the same as a missense allele. It would be more accurate to refer rme-4(b1001) as a strong loss of function or putative null.

*We have rephrased accordingly (see **line 197**).*

3. The paper does not clearly discuss limitations of the hypomorphic agef-1[S784L] and that the observed phenotypes in this hypomorph might underestimate the complete role of AGEF-1a.

*Please see **Reviewer 2, major point 2**.*

© 2026. Published by The Company of Biologists under the terms of the Creative Commons Attribution License (https://creativecommons.org/licenses/by/4.0/).

4. In figure 1, where there really only one extrachromosomal transgenic line for some of the construct tested?

We have updated Table S2 to include all tested lines.

5. The concentrations of transgenes vary in different transgenes. Is there a rationale behind this?

Yes, we have attempted multiple concentrations of injections for each transgene and there was some variability for each construct injected, thus we only included the ones where we observed an effect. We have updated Table S2 to include all tested lines.

6. In Fig.1e: I may be useful to also show the "WT" phenotype, i.e. the strong defects to get a visual comparison for the degree of rescue.

We added a representative dashed line showing the WT phenotype in ***Figure 1E*** *and* ***Figure 2B****.*

Reviewer #4 (Significance (Required)):

The study has identified AGEF-1a as a regulator of axonal maintenance, functioning to protect neurons against mechanical stress by acting through RAB-35. Additionally, this epidermal GEF, AGEF-1a is functionally conserved as its human orthologue BIG2 can replace AGEF-1a in C. elegans for axonal protection. Important points here are that the findings extend prior work by the authors of non-autonomous mechanism that regulates epidermal-neuronal attachment. In my humble opinion, the human disease connection, in particular with regard to the unexplained neuronal phenotypes in patients could be better developed in the manuscript. It may also increase impact/interest of a wonderful story that right now reads a bit 'wormy'.

We have elaborated more on how we think AGEF-1 might play a role in human diseases, such as periventricular heterotropia and spectrinopathies and where possible, we attempted to use a more accessible language and explanations.

Description of analyses that authors prefer not to carry out:

Reviewer #1

"…studies to test potentially physical interaction such as pull-down assays, co-IP experiments and FRET could be used to test whether AGEF-can bind RAB-35 or ARF-5."

While pull-down assays, co-IP and FRET would reveal whether AGEF-1 can form a complex with RAB-35, we believe that using a full length AGEF-1a would not only represent a significant technical challenge but would also not prove a direct interaction in a physiological context.

"…An optional experiment would be to look at the colocalization of RAB-35 with a known effector in wild type and agef-1(vd92) with the expectation that there would be a higher level of colocalization in agef-1 mutants. Effector pull-down experiments or perhaps a cell based GEF assay could be used (PMID: 35196081)."

We think that screening for the relevant rab-35 effector in this context and/or doing effector pull-down/cell-based GEF assays would be a significant technical challenge. We propose to address this concern by tempering our claim as suggested by the Reviewer.

"…It may be interesting to also test other aspects such as axonal deformities including swellings and vesiculation in other parts of the nervous system. Moreover, behavioral or functional experiments such as response to gentle touch or synaptic integrity could be informative."

We find these are interesting ideas that would certainly answer how the function of these neurons might be affected, but would not provide further insights into how their integrity is maintained. We believe this is beyond the scope of this story.

© 2026. Published by The Company of Biologists under the terms of the Creative Commons Attribution License (https://creativecommons.org/licenses/by/4.0/).

Second decision letter

MS ID#: jcs.264565R1

MS TITLE: A conserved Arf-GEF modulates axonal integrity through RAB-35 by altering neuron-epidermal attachment

AUTHORS: Igor Bonacossa-Pereira; Dat Le; Sean Coakley; Massimo Hilliard

ARTICLE TYPE: Review Commons Transfer

Dear Dr Hilliard,

The reviewers are enthusiastic about your work and have comments regarding clarification regarding the language used, consideration of previous studies, and further clarification of some experiments (which some readers may also have). This revision can be handled at an editorial level without sending the paper back to reviewers. Could you address their points and send me a revised version? Please also add very brief responses to the reviewers comments, especially if you choose not to incorporate their suggestions. I look forward to seeing the revised manuscript.

Reviewer 1

SUMMARY OF THE ADVANCE MADE IN THIS PAPER AND ITS POTENTIAL SIGNIFICANCE TO THE FIELD

SUGGESTIONS TO AUTHORS

The authors have mostly addressed my concerns. Their elucidation of the AGEF-1(S784L) allele as loss-of-function is nicely done, including the use of tissue-specific depletion. I will not reiterate all my enthusiastic comments from the first round of review, but this is an excellent genetic study and I am highly enthusiastic.

However, in my view, the claim that "a subset of AGEF-1 [molecules] physically interact with RAB-35" remains poorly supported:
* First, the super-resolution imaging is a nice addition but it seems to show that most AGEF-1 puncta are adjacent to, but NOT overlapping with, RAB-35. Is the image in Fig. 3D a single optical section (approximately what thickness?) or a projection?
* Second, in principle the GFP complementation assay measures proximity, not direct interaction, and would require tempering the claim of physical interaction.
* Third, I found the results of the GFP complementation assay to be confusing and unconvincing. GFP1-10 has intrinsic affinity for GFP11, which is why split-GFP is standardly used for cell-specific labeling of endogenously GFP11-tagged proteins (as in Fig. 3B-C and Supp. Fig. S2C). How does this intrinsic affinity affect the interpretation of apparent binding? It would be helpful to see the controls with AGEF-1:GFP11 + GFP1-10 alone, or AGEF-1(S784L):GFP11 + GFP1-10 alone, in Fig. 3F. These data are shown in Supp. Fig. 2C and, comparing them with Fig. 3F, there appears to be a substantial decrease in AGEF-1:GFP11 binding to GFP1-10 when it is fused to RAB-35 (from ~35 units to ~10 units; i.e., fusing GFP1-10 to RAB-35 decreases its binding to AGEF-1:GFP11). This is confusing to me, and suggests that RAB-35 impairs the ability of GFP1-10 to bind AGEF-1:GFP11. Adding to this confusion, it appears in Fig. 3B-C and Supp. Fig. 2C that there is less overall expression/availability of AGEF-1(S784L):GFP11 than AGEF-1:GFP11, which additionally complicates the interpretation of GFP-complementation data, and highlights the need for showing the controls side-by-side. In short, this assay does not seem to be measuring AGEF-1:RAB-35 affinity, but rather the intrinsic affinity of GFP1-10:GFP11 that is being impaired by fusion to proteins that mostly do not co-localize/interact. It seems likely that fusion to GFP1-10 and GFP11 is forcing an interaction between AGEF-1 and RAB-35.
* As minor issues, please include a scale bar for the intensity heat map applied to Fig. 3E (if possible, it would help to present these data the same as the GFP intensities shown in Fig. 3B-C); clarify in the legend if images are projections or optical sections; and please review the y-axis labeling of Fig. 3F (appears to be in units of particles x area, instead of particles/area).

© 2026. Published by The Company of Biologists under the terms of the Creative Commons Attribution License (https://creativecommons.org/licenses/by/4.0/).

* Many of these issues can be mitigated by softening the claims in the text.

As a minor point, the reference in the Abstract to "periventricular nodular heterotopia" seems out of place. I realize this was included in response to a Reviewer comment, but the term does not appear again anywhere in the manuscript. Is this the same as familial microcephaly, which appears in the Discussion? It makes sense to highlight disease relevance in the Abstract, but this should be consistent with the main text and should be connected to the human protein BIG2, not the C. elegans protein AGEF-1.

Reviewer 2

SUMMARY OF THE ADVANCE MADE IN THIS PAPER AND ITS POTENTIAL SIGNIFICANCE TO THE FIELD

The authors have addressed my previous concerns particularly around physical interactions between AGEF-1 and RAB-35 and not suggesting that AGEF-1 is a RAB-35 GEF in the absence of a GEF assay. I was pleased to see that AGEF-1 and RAB-35 interact via a split-GFP assay and that this interaction is disrupted by their AGEF-1 [S784L] mutation as it opens the door to some mechanism. Ideally, this would be followed up by a secondary interaction assay. I was also pleased to see a role for ARF-5. These experiments have improved the findings.
My only concerns regard 1) relating the findings with functional studies with yeast Sec7p and 2) the wording around GTPase activity and the functions of GEFs and GAPs (this was a concern of another reviewer which was not adequately addressed).
1). Yeast Sec7p is the founding member of the AGEF-1/BIG2 GEFs and there has been a lot of functional studies by the Fromme lab (I am not him) that are relevant to this study and are largely ignored.
Lines 126, 154 and 173: The HDS1 domain is defined as an auto-inhibitory domain. HDS is an acronym for Homology Downstream of Sec7. While HDS2-4 of yeast Sec7p were shown to have an autoinhibitory function, the HDS1 domain was shown to promote Sec7p GEF activity (PMID: 22516198).
Fig S1A: Would this structure correspond to the open or closed state? A recent paper reported the structure of yeast Sec7p. The determined structure revealed an autoinhibited state while Alphafold predicted the open state (PMID: 38416685). I am not sure that the Alphafold structure of AGEF-1 is adding much here unless it can be combined with the Sec7 structure to provide more information on how the S784L mutation could perturb function.
Fig 3: The AGEF-1 S784L mutation reduces the in vivo interaction with RAB-35. The HDS1 of Sec7p was shown to interact with Arf1 (PMID: 25220393). I was surprised that AGEF-1 S784L mutant wasn't also tested with ARF-1.
In reference to AGEF-1 interacting with RAB-35 it is worth mentioning that other Rab GTPases were identified as regulators of Sec7p (PMID: 25220393).

2.) There are issues with language regarding the GTPase cycle.
Line 81: "GAPs terminate GTPase activity by catalyzing GTP hydrolysis" Small GTPases are confusing in that they are enzymes that hydrolyze GTP to GDP, but they are in an active state when bound to GTP and can interact with effector proteins. Perhaps saying something like GAPs stimulate the intrinsic GTPase activity and terminate effector binding.
Line 83: "GEFs initiate GTPase activity by catalyzing the release of GDP and allowing GTP binding." Here this sounds like the GEF is playing the role of a GAP. I would suggest: GEFs promote the active state by stimulating the release of GDP…
Line 312: "we propose that mutations in BIG2 affecting its GTPase function" The way it is written it implies that BIG2 is a GTPase.
Line 314: "still dependent on its GTPase activating function." Again implying that AGEF-1 is GAP rather than a GEF."
Corrections
Line 27: Sounds like mutations in AGEF-1 are associated with periventricular heterotopia rather than BIG2.
Line 89 and 92: I think you are referring to FLCN-1, not FCNL-1.
Line 102: "we show that AGEF-1 interacts with RAB-35 and regulates its activity in vivo" The activity of RAB-35 is not actually tested but is inferred by it being downstream. Keep in mind that rescue by RAB-35 OE could simply be regulating a bypass pathway.
Line 158: E806K or E608K

© 2026. Published by The Company of Biologists under the terms of the Creative Commons Attribution License (https://creativecommons.org/licenses/by/4.0/).

Line 226: CRISPR not CRIPSR
Line 214 and 326: It is stated that ARF-5 is upstream of RAB-35 but this has not been tested.
Line396: milliQ not miliQ
Line 676: "Solid lines indicate directly validated gene interactions; dashed lines indicate proposed genetic interactions based on experimental data." It is unclear what the difference is between validated gene interactions and genetic interactions based on experimental data.
Line 702: would "colocalization" rather than "interaction" be more appropriate.
Minor comment: I like that the disease connection was made with BIG2. I realize that the focus is on BIG2 as this was demonstrated to rescue AGEF-1. However, the paralogous BIG1 is also mutated in neurodevelopmental disorders. You may consider including this for the sake of comprehensiveness.

Reviewer 3

SUMMARY OF THE ADVANCE MADE IN THIS PAPER AND ITS POTENTIAL SIGNIFICANCE TO THE FIELD

SUGGESTIONS TO AUTHORS

The authors have generally responded very well to my suggestions for improvements, including with nice new experimental results. For example, the bimolecular complementation assays with split GFP make a strong argument that AGEF-1 and RAB-35 are in very close proximity, enough to allow complementation of the GFP pieces. I am not sure, however, whether these experiments "demonstrate" that the two proteins physically interact (e.g. L259). The authors may want to tone down this conclusion a bit to "consistent with the interpretation that they physically interact" or "that they are part of the same complex". Something along these lines.

Second revision

Author response to reviewers' comments

Reviewer 1: The authors have mostly addressed my concerns. Their elucidation of the AGEF-1(S784L) allele as loss-of-function is nicely done, including the use of tissue-specific depletion. I will not reiterate all my enthusiastic comments from the first round of review, but this is an excellent genetic study and I am highly enthusiastic.

However, in my view, the claim that "a subset of AGEF-1 [molecules] physically interact with RAB-35" remains poorly supported:
* First, the super-resolution imaging is a nice addition but it seems to show that most AGEF-1 puncta are adjacent to, but NOT overlapping with, RAB-35. Is the image in Fig. 3D a single optical section (approximately what thickness?) or a projection?

We thank the reviewer for this comment. The image in Fig. 3D is a z-projection of a 750 nm of deep stack of 5 slices. In this stack, AGEF-1 overlaps with RAB-35 throughout all slices (see line scans on the panel below). The selected image is representative of 7 biological replicates. We think this image accurately reflects *lines 239-242:*

“While AGEF-1 and RAB-35 appeared largely not co-localized, we noted that a subset of epidermal AGEF-1::GFP_{x7} puncta decorated mScarlet3::RAB-35 structures; super-resolution imaging of such regions revealed that AGEF-1::GFP_{x7} overlaps with the edge of these mScarlet3::RAB-35 circular structures (Fig. 3D).”

© 2026. Published by The Company of Biologists under the terms of the Creative Commons Attribution License (https://creativecommons.org/licenses/by/4.0/).

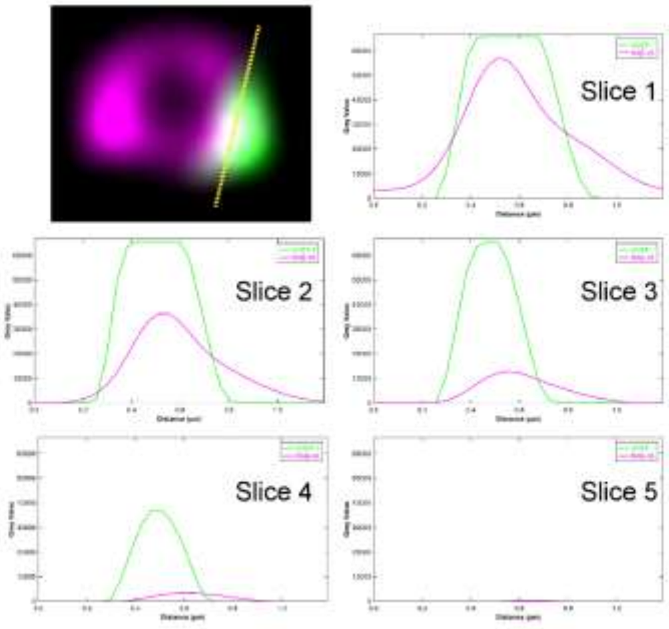

* Second, in principle the GFP complementation assay measures proximity, not direct interaction, and would require tempering the claim of physical interaction.

We agree with your comment and have changed the text to reflect this idea on *lines 250, 329, 338.*

* Third, I found the results of the GFP complementation assay to be confusing and unconvincing. GFP1-10 has intrinsic affinity for GFP11, which is why split-GFP is standardly used for cell-specific labeling of endogenously GFP11-tagged proteins (as in Fig. 3B-C and Supp. Fig. S2C). How does this intrinsic affinity affect the interpretation of apparent binding? It would be helpful to see the controls with AGEF-1:GFP11 + GFP1-10 alone, or AGEF-1(S784L):GFP11 + GFP1-10 alone, in Fig. 3F. These data are shown in Supp. Fig. 2C and, comparing them with Fig. 3F, there appears to be a substantial decrease in AGEF-1:GFP11 binding to GFP1-10 when it is fused to RAB-35 (from ~35 units to ~10 units; i.e., fusing GFP1-10 to RAB-35 decreases its binding to AGEF-1:GFP11). This is confusing to me, and suggests that RAB-35 impairs the ability of GFP1-10 to bind AGEF-1:GFP11. Adding to this confusion, it appears in Fig. 3B-C and Supp. Fig. 2C that there is less overall expression/availability of AGEF-1(S784L):GFP11 than AGEF-1:GFP11, which additionally complicates the interpretation of GFP-complementation data, and highlights the need for showing the controls side-by-side. In short, this assay does not seem to be measuring AGEF-1:RAB-35 affinity, but rather the intrinsic affinity of GFP1-10:GFP11 that is being impaired by fusion to proteins that mostly do not co-localize/interact. It seems likely that fusion to GFP1-10 and GFP11 is forcing an interaction between AGEF-1 and RAB-35.

This is indeed a complex experiment and deserves a clearer explanation. In principle, the natural affinity between GFP_{11} and GFP_{1-10} could induce a synthetic interaction. However, in this scenario, we would expect that the localization of both molecules bound to GFP_{1-10} and GFP_{11} would match the endogenous localization of one (or both). We did not observe this. In fact, for AGEF-1 and RAB-35 we observed puncta that closely resemble the regions where we see overlap between AGEF-1::GFP_{x7} and mScarlet3::RAB-35 (compare Fig. 3E with Fig. 3B-D). Further supporting this notion is our observation that the S784L mutation abolished this interaction.

In Fig. S2C, cytosolic GFP_{1-10} is overexpressed from an extrachromosomal array and cannot be directly compared to Fig 3F. In this system, the rate-limiting factor for puncta formation is the endogenous AGEF-1::GFP_{11x7} expression level, and therefore the decrease seen in the S784L mutant likely represents a decrease in the endogenous levels of this mutated protein rather than its ability to bind GFP_{1-10} (which is overexpressed). In contrast, in Fig. 3F, we are testing if the endogenous molecules interact with enough proximity to allow GFP reconstitution. Here, the rate-limiting factor for puncta formation is the balance of the expression level of each binding partner. In this

© 2026. Published by The Company of Biologists under the terms of the Creative Commons Attribution License (https://creativecommons.org/licenses/by/4.0/).

system, we are only able to reveal subsets of AGEF-1::GFP_{11x7} that are in proximity to either GFP_{1-10}::ARF-1 or GFP_{1-10}::RAB-35. Naturally, the number of puncta observed in these conditions will be fewer than what is observed in Fig. 3B, C and S2C. We have revised the manuscript to include a clearer explanation of our interpretation on *lines 253-267* and have included the control images in Fig. 3E.

* As minor issues, please include a scale bar for the intensity heat map applied to Fig. 3E (if possible, it would help to present these data the same as the GFP intensities shown in Fig. 3B-C); clarify in the legend if images are projections or optical sections; and please review the y-axis labeling of Fig. 3F (appears to be in units of particles x area, instead of particles/area).

Thanks for this suggestion, we have changed the LUT to match the green LUT in Fig. 3B-D and revised the plot labelling.

* Many of these issues can be mitigated by softening the claims in the text.

As a minor point, the reference in the Abstract to "periventricular nodular heterotopia" seems out of place. I realize this was included in response to a Reviewer comment, but the term does not appear again anywhere in the manuscript. Is this the same as familial microcephaly, which appears in the Discussion? It makes sense to highlight disease relevance in the Abstract, but this should be consistent with the main text and should be connected to the human protein BIG2, not the C. elegans protein AGEF-1.

Thanks for noting this. Both terms refer to the same condition, and we have revised the text to use a consistent nomenclature.

Reviewer 2: The authors have addressed my previous concerns particularly around physical interactions between AGEF-1 and RAB-35 and not suggesting that AGEF-1 is a RAB-35 GEF in the absence of a GEF assay. I was pleased to see that AGEF-1 and RAB-35 interact via a split-GFP assay and that this interaction is disrupted by their AGEF-1 [S784L] mutation as it opens the door to some mechanism. Ideally, this would be followed up by a secondary interaction assay. I was also pleased to see a role for ARF-5. These experiments have improved the findings.
My only concerns regard 1) relating the findings with functional studies with yeast Sec7p and 2) the wording around GTPase activity and the functions of GEFs and GAPs (this was a concern of another reviewer which was not adequately addressed).
1). Yeast Sec7p is the founding member of the AGEF-1/BIG2 GEFs and there has been a lot of functional studies by the Fromme lab (I am not him) that are relevant to this study and are largely ignored.
Lines 126, 154 and 173: The HDS1 domain is defined as an auto-inhibitory domain. HDS is an acronym for Homology Downstream of Sec7. While HDS2-4 of yeast Sec7p were shown to have an autoinhibitory function, the HDS1 domain was shown to promote Sec7p GEF activity (PMID: 22516198).
Fig S1A: Would this structure correspond to the open or closed state? A recent paper reported the structure of yeast Sec7p. The determined structure revealed an autoinhibited state while Alphafold predicted the open state (PMID: 38416685). I am not sure that the Alphafold structure of AGEF-1 is adding much here unless it can be combined with the Sec7 structure to provide more information on how the S784L mutation could perturb function.
Fig 3: The AGEF-1 S784L mutation reduces the in vivo interaction with RAB-35. The HDS1 of Sec7p was shown to interact with Arf1 (PMID: 25220393). I was surprised that AGEF-1 S784L mutant wasn't also tested with ARF-1.
In reference to AGEF-1 interacting with RAB-35 it is worth mentioning that other Rab GTPases were identified as regulators of Sec7p (PMID: 25220393).

We thank the reviewer for the suggestions. We now properly define the term HDS1 as suggested on line 127. The function of the HDS1 is complex, and it has been proposed that it functions both to promote Sec7p GEF activity and serve as an autoinhibitory domain (PMID: 22516198, PMID:25220393). To better communicate the known functions of the HDS1 domain more accurately we have revised the manuscript to refer to it as a regulatory domain. Moreover, we have added the relevant citations to the manuscript on *lines 125, 156, 335.*

© 2026. Published by The Company of Biologists under the terms of the Creative Commons Attribution License (https://creativecommons.org/licenses/by/4.0/).

The AlphaFold model of AGEF-1 structure corresponds to the open state as reported in PMID: 38416685. Considering these suggestions, we updated this panel (Fig S1A), to show the predicted binding site between AGEF-1 and RAB-35 as well as the Sec7 and HDS1 domains. We think it is a valid addition to the manuscript and helps the reader situate where the S784L mutation is relative to the catalytic site. We think that having it as a supplemental figure is appropriate.

We attempted to test the interaction between AGEF-1[S784L] and ARF-1, however the resulting strain was lethal. We have added a sentence in *lines 263-265* to communicate this observation.

2.) There are issues with language regarding the GTPase cycle.
Line 81: "GAPs terminate GTPase activity by catalyzing GTP hydrolysis" Small GTPases are confusing in that they are enzymes that hydrolyze GTP to GDP, but they are in an active state when bound to GTP and can interact with effector proteins. Perhaps saying something like GAPs stimulate the intrinsic GTPase activity and terminate effector binding.
Line 83: "GEFs initiate GTPase activity by catalyzing the release of GDP and allowing GTP binding." Here this sounds like the GEF is playing the role of a GAP. I would suggest: GEFs promote the active state by stimulating the release of GDP…
Line 312: "we propose that mutations in BIG2 affecting its GTPase function" The way it is written it implies that BIG2 is a GTPase.
Line 314: "still dependent on its GTPase activating function." Again implying that AGEF-1 is GAP rather than a GEF." Replaced all occurrences of GTPase activating function with GEF function.
Corrections
Line 27: Sounds like mutations in AGEF-1 are associated with periventricular heterotopia rather than BIG2.
Line 89 and 92: I think you are referring to FLCN-1, not FCNL-1.
Line 102: "we show that AGEF-1 interacts with RAB-35 and regulates its activity in vivo" The activity of RAB-35 is not actually tested but is inferred by it being downstream. Keep in mind that rescue by RAB-35 OE could simply be regulating a bypass pathway.
Line 158: E806K or E608K
Line 226: CRISPR not CRIPSR
Line 214 and 326: It is stated that ARF-5 is upstream of RAB-35 but this has not been tested.
Line396: milliQ not miliQ
Line 676: "Solid lines indicate directly validated gene interactions; dashed lines indicate proposed genetic interactions based on experimental data." It is unclear what the difference is between validated gene interactions and genetic interactions based on experimental data.
Line 702: would "colocalization" rather than "interaction" be more appropriate.

We thank the reviewer for the suggestions, and have revised the text accordingly.

Minor comment: I like that the disease connection was made with BIG2. I realize that the focus is on BIG2 as this was demonstrated to rescue AGEF-1. However, the paralogous BIG1 is also mutated in neurodevelopmental disorders. You may consider including this for the sake of comprehensiveness.

We have included a sentence in *lines 315-317* and revised naming from BIG2 to BIG1/2 in *lines 317-321* to discuss this concept.

Reviewer 3: SUMMARY OF THE ADVANCE MADE IN THIS PAPER AND ITS POTENTIAL SIGNIFICANCE TO THE FIELD

SUGGESTIONS TO AUTHORS
The authors have generally responded very well to my suggestions for improvements, including with nice new experimental results. For example, the bimolecular complementation assays with split GFP make a strong argument that AGEF-1 and RAB-35 are in very close proximity, enough to allow complementation of the GFP pieces. I am not sure, however, whether these experiments "demonstrate" that the two proteins physically interact (e.g. L259). The authors may want to tone down this conclusion a bit to "consistent with the interpretation that they physically interact" or "that they are part of the same complex". Something along these lines.

© 2026. Published by The Company of Biologists under the terms of the Creative Commons Attribution License (https://creativecommons.org/licenses/by/4.0/).

Thanks for the suggestion. We have changed *lines 265-267* to reflect this notion and tempered this claim in all instances.
****

Third decision letter

MS ID#: jcs.264565R2

MS Title: A conserved Arf-GEF modulates axonal integrity through RAB-35 by altering neuron-epidermal attachment

Authors: Igor Bonacossa-Pereira; Dat Le; Sean Coakley; Massimo Hilliard

Article Type: Review Commons Transfer

Dear Dr Hilliard,

I am happy to tell you that your manuscript has been accepted for publication in Journal of Cell Science, pending standard publication integrity checks.

Thank you for sending your manuscript to Journal of Cell Science through Review Commons.

© 2026. Published by The Company of Biologists under the terms of the Creative Commons Attribution License (https://creativecommons.org/licenses/by/4.0/).
